# Increased Vascular Contractility in Hypertension Results From Impaired Endothelial Calcium Signaling

**DOI:** 10.1161/HYPERTENSIONAHA.119.13791

**Published:** 2019-09-23

**Authors:** Calum Wilson, Xun Zhang, Charlotte Buckley, Helen R. Heathcote, Matthew D. Lee, John G. McCarron

**Affiliations:** From the Strathclyde Institute of Pharmacy and Biomedical Sciences, University of Strathclyde, Glasgow, United Kingdom.

**Keywords:** blood pressure, calcium, endothelial cells, hypertension, phenylephrine

## Abstract

Endothelial cells line all blood vessels and are critical regulators of vascular tone. In hypertension, disruption of endothelial function alters the release of endothelial-derived vasoactive factors and results in increased vascular tone. Although the release of endothelial-derived vasodilators occurs in a Ca^2+^-dependent manner, little is known on how Ca^2+^ signaling is altered in hypertension. A key element to endothelial control of vascular tone is Ca^2+^ signals at specialized regions (myoendothelial projections) that connect endothelial cells and smooth muscle cells. This work describes disruption in the operation of this key Ca^2+^ signaling pathway in hypertension. We show that vascular reactivity to phenylephrine is increased in hypertensive (spontaneously hypertensive rat) when compared with normotensive (Wistar Kyoto) rats. Basal endothelial Ca^2+^ activity limits vascular contraction, but that Ca^2+^-dependent control is impaired in hypertension. When changes in endothelial Ca^2+^ levels are buffered, vascular contraction to phenylephrine increased, resulting in similar responses in normotension and hypertension. Local endothelial IP_3_(inositol trisphosphate)-mediated Ca^2+^ signals are smaller in amplitude, shorter in duration, occur less frequently, and arise from fewer sites in hypertension. Spatial control of endothelial Ca^2+^ signaling is also disrupted in hypertension: local Ca^2+^ signals occur further from myoendothelial projections in hypertension. The results demonstrate that the organization of local Ca^2+^ signaling circuits occurring at myoendothelial projections is disrupted in hypertension, giving rise to increased contractile responses.

## Abstract 2

Supplemental Digital Content is available in the text.

Hypertension is a chronic condition that contributes to the development of numerous cardiovascular diseases, including heart failure, vascular dementia, and stroke. As raised blood pressure levels lead to 7.6 million premature deaths each year (≈13.5% of the global total),^1^ hypertension contributes to worldwide morbidity and mortality more than any other risk factor. Although increased blood pressure levels are positively and continuously related to increasing cardiovascular risk, the precise mechanisms that lead from hypertension to ill health are poorly understood. One reason for this poor understanding is that the pathophysiology of hypertension is complex, with contributions from the sympathetic nervous system, the renin-angiotensin-aldosterone system, and the immune system each implicated in its cause.^2–5^ Notwithstanding, the adverse effects of hypertension are mediated by changes in the structure and function of the artery wall.^6–8^ Functional changes, in the form of altered vascular reactivity, occur at the earliest stages of hypertension and contribute to the development and reinforcement of the condition. However, the precise mechanisms contributing to altered vascular reactivity are not well understood.

In healthy blood vessels, the endothelial cell lining of blood vessels (the endothelium) controls vascular reactivity (and hence blood pressure) by releasing paracrine signaling molecules, such as nitric oxide (NO) and prostacyclin. The activation of endothelial potassium channels may also initiate a hyperpolarization of the plasma membrane that spreads to neighboring smooth muscle cells.^9^ These endothelial signaling pathways normally act to attenuate vascular contraction or promote vasodilation or both,^10–15^ but their action is diminished in several cardiovascular pathophysiologies. The reduced modulatory influence—termed endothelial dysfunction—is, at least in part, responsible for dysfunctional vascular responses (increased contractile and decreased dilator responses), which accompany and aggravate chronic elevations in blood pressure.^16,17^

The production of endothelial-derived vasoactive factors and activation of endothelial potassium channels require elevations in intracellular Ca^2+^ levels. Disruption of endothelial Ca^2+^ signaling may thus lead to dysfunctional vascular responses. However, little is known on the regulation of endothelial Ca^2+^ signaling in hypertension. In cell culture models, agonist-evoked endothelial Ca^2+^ responses throughout the cells may increase,^16^ decrease,^18,19^ or be unaltered^9,17^ in hypertension. Muscarinic receptor–mediated, global Ca^2+^ signals are similar in native endothelial cells of hypertensive and normotensive mice.^20^ However, local signals arising from TRPV4 (transient receptor potential vanilloid 4)-mediated Ca^2+^ influx are reduced.^20^ This latter observation demonstrates that disruption of local Ca^2+^ signaling circuits, rather than global increases in Ca^2+^, may contribute to endothelial dysfunction in hypertension.^20^

Endothelial IP_3_(inositol trisphosphate) receptors play an essential role in regulating blood pressure.^21,22^ Indeed, most physiological endothelial cell stimuli exert their effects by triggering the production of IP_3_. Once produced, IP_3_ binds to IP_3_Rs (IP_3_ receptors) to cause the release of Ca^2+^ from intracellular stores. Localized IP_3_-mediated Ca^2+^ activity occurs spontaneously in unstimulated endothelium^23^ and is amplified by extracellular agonists to limit basal and activated smooth muscle tone.^24^ Of particular significance in endothelial control of vascular tone are Ca^2+^ signals that occur preferentially at sites where endothelial cells protrude through the internal elastic lamina (IEL) and contact smooth muscle cells (myoendothelial projections, MEPs).^23^ MEPs are packed with Ca^2+^-activated effector proteins, including eNOS (endothelial NO synthase)^18^ and the small and intermediate conductance Ca^2+^-sensitive potassium channels,^19^ creating pivotal microdomains that are critical to the regulation of vascular function. Yet, it is unknown if local IP_3_-mediated Ca^2+^ signaling at MEPs is altered in hypertension.

Here, we investigated whether disruption of local, IP_3_-mediated, endothelial Ca^2+^ signaling is responsible for the hypercontractile smooth muscle cell responses that occur in hypertension. A novel technique was used to simultaneously assess the vascular contractile state and endothelial Ca^2+^ levels in intact small mesenteric arteries. We show that basal IP_3_-mediated endothelial Ca^2+^ signaling opposes vascular tone in arteries from normotensive (Wistar Kyoto; WKY) and hypertensive rats (spontaneously hypertensive rat; SHR). However, the extent to which endothelial Ca^2+^ signaling limits vascular tone is significantly reduced in hypertension. We also found that the occurrence and amplitude of local IP_3_-mediated Ca^2+^ signaling events are reduced in hypertension. Significantly, the distance between local Ca^2+^ signals and MEPs is increased. Together, these results suggest disruption of local IP_3_-mediated Ca^2+^ signaling at MEPs explains the impaired endothelial control of vascular tone and the increased contractile state of vascular smooth muscle in hypertension.

## Materials and Methods

The data that support the findings of this study are available from the corresponding authors on reasonable request.

### Animal Model of Hypertension

All animal care and experimental procedures were conducted in accordance with relevant guidelines and regulations with the approval of the University of Strathclyde Local Ethical Review Panel, under UK Home Office regulations (Animals [Scientific Procedures] Act 1986, United Kingdom). Eight- to 10-week-old male SHR (n=16) and WKY (n=16) rats purchased from Envigo (United Kingdom) were housed in pairs (with a reversed 12-hour light/dark cycle) and allowed ad libitum access to standard rat chow and water. The animals were housed 3 per cage, and the cage type was North Kent Plastic model RC2F with nesting material Sizzle Nest. A 12:12 light/dark cycle was used with a temperature range of 19 to 23°C (set point 21°C) and humidity levels between 45% and 65%. Animals had free access to freshwater and SDS diet RM1 (rodent maintenance). The enrichment in the cages was aspen wood chew sticks and hanging huts. Male rats are a widely-used experimental model of hypertension with a wealth of background information to aid interpretation of results. All animals were acclimatized for a 1-week period. After the acclimatization period, blood pressure readings were taken twice a week over a 3-week period by tail-cuff plethysmography. Mean blood pressure in normotensive (WKY) rats was 122±3 mm Hg, whereas that in hypertensive rats (SHR) was 173±6 mm Hg. All rats were euthanized by cervical dislocation at 10 to 12 weeks of age (250–350 g). Controls and experimental treatments were performed in the same tissue, so blinding and randomization were not used.

### En Face Artery Preparation

Immediately following euthanasia, the mesenteric bed was removed and placed in physiological saline solution composed (in mM) of 145 NaCl, 4.7 KCl, 2.0 (3-[N-morpholino]propanesulfonic acid (MOPS)), 1.2 NaH_2_PO_4_, 5.0 glucose, 2.0 pyruvate, 0.02 EDTA, 1.17 MgCl_2_, 2.0 CaCl_2_, adjusted to pH 7.4 with NaOH. Second- or third-order mesenteric arteries (<250 μm outer diameter) were then cleaned of connective tissue and fat, removed from the mesenteric bed, cut open using microscissors and pinned endothelial side up either (1) on the Sylgard-coated base of a custom microscope chamber designed for use on an upright microscope or (2) on a Sylgard block that was subsequently inverted and placed in a custom chamber designed for use on an inverted microscope.^25–27^ Endothelial cells, which lie parallel to the longitudinal axis of the artery, were loaded with the fluorescent Ca^2+^ indicator, Cal-520/AM (5 µmol/L with 0.04% Pluronic F127 and 0.26% dimethyl sulfoxide [DMSO] in physiological saline solution [PSS]) at 37^°^C for 30 minutes. After incubation, preparations were gently washed in PSS and mounted on a microscope for imaging. Smooth muscle cells, which lie underneath and perpendicular to endothelial cells, were not significantly loaded with Cal-520/AM, as indicated by an absence of fluorescence signal arising from the staining of cells lying perpendicular to the longitudinal axis of the artery.

### Assessment of Vascular Reactivity

Intact arteries, pinned on a Sylgard base of a custom chamber, were gently stretched to ≈1.5× resting width to prevent intimal folding. Pinning was restricted to the outermost corners. When applied to mesenteric arteries, this level of stretch results is equivalent to that induced by a transmural pressure of 80 mm Hg.^28,29^ The central region of the artery was not pinned to allow contraction to occur freely. An upright fluorescence microscope (FN-1; Nikon, Tokyo, Japan) was used to image the endothelium. Cal-520/AM was excited with 488-nm light using a wide-field epifluorescence light-emitting diode (LED) illumination system (pE-4000; CoolLED, Andover, United Kingdom), and fluorescence emission was imaged at 10 Hz using a ×16 objective lens (0.8 numerical aperture [NA]; Nikon, Tokyo, Japan) and a large-format (1024×1024·13 µm pixels) back-illuminated electron-multiplying charge-coupled device camera (iXon 888; Andor, Belfast, United Kingdom). The resulting large field-of-view (832×832 µm) was sufficient to view the edges of arteries of up to ≈250 µm passive diameter. To measure contraction, equivalent diameter was calculated from the opened artery by edge detection using a custom algorithm written in Python (Figure S1 in the online-only Data Supplement). First, 16-bit images were enhanced by custom-written preprocessing macros using the ImageJ-based open-source image processing package, FIJI.^30^ The preprocessing steps were as follows: (1) image stacks were temporally smoothed (to reduce noise) using a 10-frame (1 s) running average; (2) images were then spatially smoothed (also to reduce noise) by convolving images with a gaussian filter of 10-pixel SD; (3) image stacks were then converted to 8-bit using a linear contrast enhancement that scaled all values between the stack minima and maxima to the range 0 to 255.

Intensity profiles (used to determine the artery edge) were measured along a scan line perpendicular to the longitudinal axis of the artery by a custom Python analysis script using the profile-line function of the scikit-image processing library.^31^ These intensity profiles plot signal intensity as a function of position along the scanline. The edges of an artery with fluorescently labeled endothelia are readily identified by rapid changes in the intensity function from background to endothelial fluorescence levels and vice versa (Movie S1 and Figure S1). To determine the position of the artery edges, the derivative of smoothed (251-point, fifth-order Savitzky-Golay filter) intensity profiles was obtained. The artery edges correspond to extrema in the first derivative, which was identified using a zero-crossing detector.^25^ From the artery edge positions, we calculated the width of the en face artery preparation and transformed this width (equal to the unfolded circumference of the intact artery) to the equivalent diameter of the intact artery.

**Figure 1.**
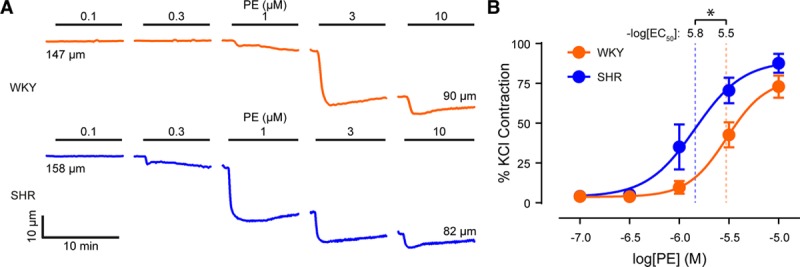
Contractions to phenylephrine (PE) are enhanced in hypertension. **A**, Representative diameter traces showing the effects of cumulatively applied phenylephrine (0.1 µmol/L–10 µmol/L) on small mesenteric arteries from normotensive (Wistar Kyoto [WKY], **top**) and hypertensive (spontaneously hypertensive rat [SHR], **bottom**) animals. **B**, Concentration-response curves for the contractile effect of phenylephrine on small mesenteric arteries. Contraction is expressed as a percentage of the maximum contraction induced by a depolarizing solution containing 70 mmol/L KCl, which did not differ between strains (Figure S3). Data are shown as mean values±SEM (n=5 for each). *Significance (*P*<0.05) using extra sum-of-squares *F* test. EC_50_ indicates half-maximal effective concentration

To validate the experimental procedure for measurement of artery contraction, synthetic image data was generated using a logistic function to describe the width of an en face preparation (Figure S1A through S1C). The algorithm generated faithful traces (Figure 1A through 1C).

### Simultaneous Assessment of Endothelial Ca^2+^ and Arterial Tone

**Figure 2.**
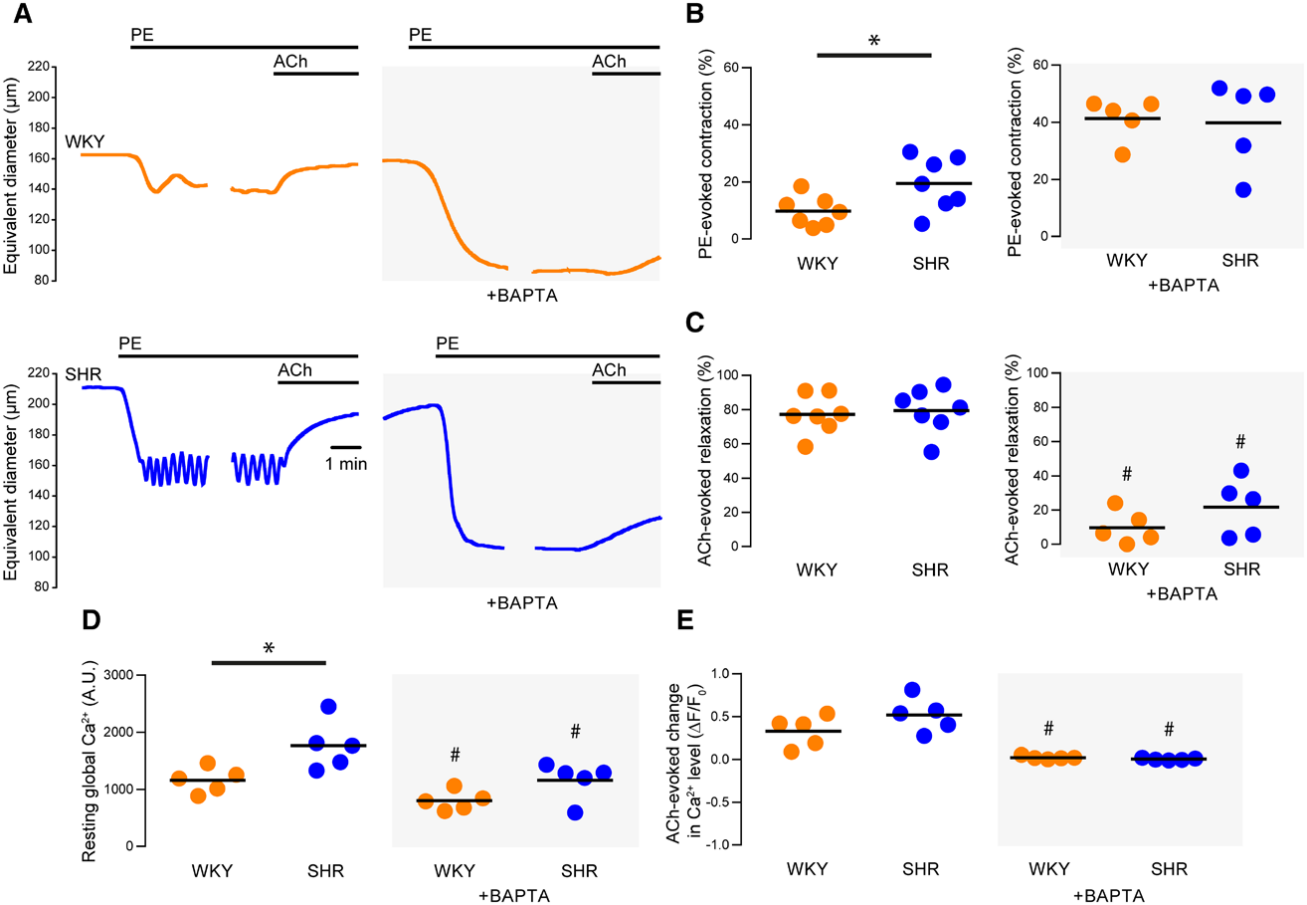
Endothelial Ca^2+^ activity is reduced by BAPTA-AM. **A**, Representative artery diameter traces from normotensive (Wistar Kyoto [WKY], **top**) and hypertensive (spontaneously hypertensive rat [SHR], **bottom**) animals before (**left**) and after (**right**) buffering of endothelial Ca^2+^ using 1,2-Bis(2-aminophenoxy)ethane-N,N,N′,N′-tetraacetic acid tetrakis acetoxymethyl ester. Phenylephrine (PE) and acetylcholine (ACh) were each used at a concentration of 1 µmol/L. **B** and **C**) Summary data showing PE-induced contraction (**B**) and ACh-induced dilation (**C**) before (**left**) and after (**righ**t) buffering of intracellular Ca^2+^ with BAPTA. **D**, Summary data illustrating the effects of BAPTA on resting endothelial Ca^2+^ levels in the absence and presence of BAPTA. **E**, Summary data illustrating the effects of BAPTA on ACh-evoked increases (ΔF/F_0_) in endothelial Ca^2+^ levels. ACh-evoked changes in Ca^2+^ levels were assessed before the onset of relaxation. WKY is denoted by orange data points and SHR by blue data points. *P<0.05 for WKY vs SHR, unpaired *t* test with Welch correction. #*P*<0.05 vs corresponding control, paired *t* test. A.U. indicates arbitrary units.

When stimulated by phenylephrine (1 µmol/L), en face arteries contracted and reached steady-state within a few minutes (Figure S2B and S2C, Movie S1). Vasoconstriction was always followed by a small increase in the fluorescence intensity obtained from a small region of interest (ROI) placed over the central portion of the artery. The subsequent addition of acetylcholine (1 µmol/L) evoked relaxations back to resting levels (Figure 2C, also Movie S2). Vasodilator responses were always preceded by an increase in fluorescence intensity (Figure S2C, Movie S2). However, because of movement, it was not possible to extract reliable, time-dependent Ca^2+^ traces from these data. Instead, we assessed the effects of pharmacological agents on resting Ca^2+^ levels (in the absence of any contractile agent) or on acetylcholine-evoked increases in endothelial Ca^2+^ levels which were measured before the onset of any mechanical response (Figure S2C). In some experiments to prevent Ca^2+^ changes, the endothelium was loaded with the Ca^2+^ chelator 1,2-Bis(2-aminophenoxy)ethane-N,N,N′,N′-tetraacetic acid tetrakis(acetoxymethyl ester) (BAPTA-AM) (30 μM; 0.04% Pluronic F127 and 0.26% DMSO in PSS) for 30 minutes at 37^°^C.

### High-Resolution Imaging of Endothelial Ca^2+^ Signaling

Basal endothelial Ca^2+^ activity was imaged at high temporal (20 Hz) and spatial (130 nm projected pixel size at focal plane) resolution using an inverted fluorescence microscope (TE2000U; Nikon, Tokyo, Japan) equipped with a ×100 objective (1.3 NA; Nikon, Tokyo, Japan) and a large-format (1024×1024·13 µm pixels) electron-multiplying charge-coupled device camera (iXon 888; Andor, Belfast, United Kingdom). Cal-520/AM was excited with 488-nm wide-field epifluorescence illumination provided by a monochromator (Photon Technology International/Horiba UK, Ltd, Stanmore, United Kingdom). The resulting image field (≈133 µm×133 µm) enabled visualization of connected networks of ≈50 whole or partial endothelial cells. Ca^2+^ activity was recorded for periods of 60 s and spontaneous (local) Ca^2+^ activity, which was clearly visible in raw recordings, was analyzed as described below.

In some experiments, we assessed global endothelial Ca^2+^ responses to photolysis of caged IP_3_.^32,33^ In these experiments, the endothelium was dual-loaded with Cal-520/AM (5 μM) and a membrane-permeant caged IP3, caged IP3 4,5-dimethoxy-2-nitrobenzyl (10 μM), 0.02% Pluronic F-127, and 0.35% DMSO in PSS for 30 minutes at 37°C. Endothelial Ca^2+^ imaging was then imaged at 10 Hz, using an inverted fluorescence microscope (TE300; Nikon, Tokyo, Japan) equipped with a ×40 objective (1.3 NA; Nikon, Tokyo, Japan) and a large-format (1024×1024 13-μm pixels) electron-multiplying charge-coupled device camera (iXon 888; Andor, Belfast, United Kingdom) with a 325-nm projected pixel size at focal plane. Cal-520/AM was excited with 488-nm illumination (PE-300Ultra, CoolLED, Andover, United Kingdom), and the electron-multiplying charge-coupled device FOV was restricted to the central 512×512 pixels, resulting in a field-of-view of ≈166×166 μm (>50 cells visualized). Photolysis of caged IP3 was achieved using a xenon flashlamp (Rapp Optoelectronic, Hamburg, Germany) attached directly to the epi-illuminator of a TE300 microscope.^34,35^ The photolysis spot size diameter was ≈70 μm. Identical UV flashes in the absence of caged IP_3_ evoked no detectable Ca^2+^ response.

### Automated Analysis of Local Endothelial Ca^2+^ Signaling

Local endothelial Ca^2+^ signaling was analyzed using a custom Python-based analysis suite for batch processing large numbers of datasets. The procedure for analyzing local Ca^2+^ signals consisted of 4 parts: (1) preprocessing of raw Ca^2+^ imaging data; (2) identification of sites of Ca^2+^ activity; (3) extraction of Ca^2+^ signals from active sites; (4) analysis of Ca^2+^ event parameters. To do this, we used algorithms that we have previously used to assess local endothelial Ca^2+^ signals.^25,26,36,37^ The first^37^ was specifically designed for the detection of local Ca^2+^ events in charge-coupled device (CCD) imaging data, whereas the second^26^ was designed to extract and analyze Ca^2+^ signals from a large number of ROIs. We combined these 2 algorithms to automatically generate traces of local Ca^2+^ signaling events from imaging data and, from these traces, extract temporal and spatial metrics of each Ca^2+^ event. Each step is described below.

#### Image Preprocessing

Ca^2+^ imaging recordings were preprocessed as previously described.^25^ In brief, to facilitate algorithmic detection of Ca^2+^ events, we first created image stacks representing fractional fluorescence changes (Δ*F*) by dividing each frame by the mean of all frames and subtracting a value of 1 from every pixel; the resultant stack was further processed by applying a gaussian blur (2-pixel radius). Finally, Δ*F* image stacks were converted to binary form by applying a threshold (mean pixel intensity within the stack plus 3× the SD of pixel intensity within the stack). The preprocessing resulted in a binary Δ*F* image stack where a pixel value of 1 (or 0) indicated the presence (or absence) of a Ca^2+^ event above threshold. All image preprocessing was performed using batch processing macros in FIJI.

#### Identification of Ca^2+^ Event Initiation Sites

Ca^2+^ events were identified from binary image stacks using code obtained from the detect puffs plug-in of FLIKA. This algorithm and its use for local Ca^2+^ event detection have been described extensively (eg,^38,39^). The algorithm calculates the 3-dimensional coordinates (*x*,*y*,*t*) of each unique Ca^2+^ event indicated in the binary Δ*F* stack and is optimized for detecting fast Ca^2+^ events (ie, Ca^2+^ puffs). Using the coordinates generated, the algorithm maps each Ca^2+^ event in the original data and extracts principal measurements in space (event location) and time (amplitude, rise time, fall time, full duration at half maximum [FDHM]). We optimized the FLIKA algorithm to permit detection of Ca^2+^ waves and identification of wave initiation sites and incorporated the software into our own batch processing code. In brief, FLIKA algorithms were used to generate coordinates that bounded each detected Ca^2+^ event and to generate multiple Δ*F* image stacks that each contained a single Ca^2+^ event. A spatial image indicating the initiation site of each Ca^2+^ event was then generated by taking a projection (SD of intensity) of each Δ*F* image stack of 10 frames (0.5 s) around the start of the corresponding event. The coordinates of the initiation site were calculated by fitting 2-dimensional elliptical gaussian function to this image. We repeated this process, creating a spatial image of each Ca^2+^ event in its entirety which allowed us to calculate the total area (spatial spread) of each event. The spatial spread of each event was determined by calculating the elliptical area under a fitted 2-dimensional gaussian. We used the initiation site coordinates, generated by the FLIKA algorithm, to generate ROIs (15-pixel diameter) for subsequent extraction of temporal Ca^2+^ signals from original image stacks.

#### Analysis of Ca^2+^ Event Parameters

Temporal Ca^2+^ signals were extracted from the raw fluorescence intensity (*F*) image stacks, using 15-pixel (≈2 µm) diameter circular ROIs positioned at the initiation site of each Ca^2+^ event as described above. The signals were processed using a modification of our previously published algorithm for the batch processing of 2-dimensional Ca^2+^ data.^36^ Multiple events may arise from the same site, so we considered ROIs that were separated by a distance <15 pixels (≈2 µm) to arise from a single site and the ROI corresponding to the first detected Ca^2+^ event was used. Fluorescence intensity traces were smoothed using an 11-point (0.55 s), third-order polynomial Savitzky-Golay filter, corrected for baseline drift using asymmetrical least squares fitting^40^ and differentiated by convolution with the first derivative of gaussian kernel. Fluorescence intensity (*F*) traces were then expressed as fractional changes from baseline (*F/F*_*0*_) by dividing values in the fluorescence intensity trace by the average value of a 100-frame (5 s) baseline-period (*F*_0_). The baseline period was automatically determined for each trace as the portion of signal exhibiting the lowest SD. This was achieved by applying, in turn, a rolling SD (100-frame) and a rolling summation (100-frame) to each trace. The minimum of the rolling summation corresponds to the center of the quietest portion of the *F/F*_*0*_ trace. Peaks in each *F/F*_*0*_ trace were identified using a zero-crossing detector on the corresponding derivative and a threshold of 10× the SD of baseline noise was used to distinguish Ca^2+^ events from noise. Event parameters (amplitude, FDHM, 10%–90% rise time, and 90%–10% fall time) were then extracted by fitting each detected Ca^2+^ event with a gaussian function.

### Comparison of Methods for Identifying Ca^2+^ Event Initiation Sites

Some studies question the application of automated analyses to endothelial Ca^2+^ signaling in intact arteries.^41^ Criticism of automated analysis is valid when applied to recordings with a poor signal-to-noise ratio or those that exhibit tissue movement, as each condition can introduce severe artifacts. Indeed, it is partly for this reason that we did not attempt to extract local endothelial Ca^2+^ signals from the dynamic imaging experiments where we assessed smooth muscle cell contraction. However, with suitable imaging data (ie, high signal-to-noise ratio, lack of focus drift, stage drift, or tissue movement), automated analysis is objective, removes operator bias, and permits a quantity of data to be processed that would be unmanageable by manual methods, such that statistical confidence is increased.

To validate the present approach, we directly compared results obtained using the algorithm with those obtained through manual identification of Ca^2+^ event initiation sites by an experienced operator (in a small subset of the data). Event initiation sites were identified by visual identification of *ΔF* image stacks. When an event was detected, a subcellular ROI was placed at the point of origin unless the event originated out with the field-of-view. Binary images were then generated in which each event initiation site was indicated by a white circle, and the results from each method (manual and automated) were compared. We found that the identification of Ca^2+^ event initiation sites was comparable, whether performed by manual or automated methods (Figure S2). However, the manual approach is practical with only very small (and perhaps unrepresentative) data sets and not with the large image areas and long recording times used in the present study.

### Assessment of Ca^2+^ Event Initiation Site and Myoendothelial Gap Junction Location

To assess coupling between Ca^2+^ events and MEPs, we imaged the underlying IEL at each site from which Ca^2+^ imaging data was recorded. The IEL was visualized using 390 nm light, and single images were generated by averaging 100-frame recordings obtained at 10 Hz. To highlight the position of IEL holes, we smoothed and inverted IEL images so that IEL holes were instead shown as bright regions on a dark background. IEL hole images were subjected to spatial filtering (2.5-pixel gaussian kernel) and intensity thresholding. Binary Ca^2+^ event initiation site images were created by flood filling the initiation site ROIs. IEL hole content and IEL hole/Ca^2+^ event initiation site localization was then determined using custom-written Python code. IEL holes were identified by 4-point connectivity using the measure.label function of the scikit-image processing library.^31^ The centroid-centroid distance between every Ca^2+^ event and every IEL holes was measured, and the closest IEL hole to each Ca^2+^ event initiation site determined.

To determine whether the extent of colocalization between Ca^2+^ event initiation sites and IEL holes was greater than would be expected if initiation sites and IEL holes were randomly positioned with respect to each other, we used a permutation analysis.^42^ First, existing Ca^2+^ event initiation site data were used to simulate a random distribution of initiation sites. Initiation sites within the field-of-view were randomized and the location of IEL holes left unchanged. Colocalization between the randomized Ca^2+^ event initiation sites and the unchanged IEL holes was then calculated as described above. For each dataset, this process was repeated 1000×, and a distribution of the minimum (random) initiation site to IEL hole separation calculated. To determine what fraction of Ca^2+^ event initiation sites colocalized with IEL holes, we used the randomized data to set a threshold (fifth percentile) distance for each dataset. Ca^2+^ events were considered as being localized to an IEL hole if the corresponding separation was less than this threshold.

### Data Presentation and Statistical Analysis

For studies of basal Ca^2+^ activity, data were collected from at least 3 different fields of endothelial cells from 3 different arteries per rat. In experiments examining vascular reactivity or Ca^2+^ responses to acetylcholine, ionomycin, or photolysis of caged IP_3_, data were collected from a single artery segment per rat. Except for probability distributions, the n number represents the unit of analysis (ie, number of experimental animals). To create probability distributions, data were pooled from all experimental animals within each treatment group. In general, summary data is presented graphically as individual data points (mean of means within each experimental unit) with the grand mean indicated. Non-Gaussian data (identified using the D’Agostino-Pearson omnibus test) were log-normal. Log-normal data were transformed (log_10_) and mean values for each experimental unit were calculated on the logarithmic scale and then back-transformed to their original scale for presentation. Graphically, summarized log-normal data are presented as back-transformed means of individual data points with the grand mean indicated, whereas in the text as back-transformed grand means with 95% CIs. Summary data were compared using 2-tailed *t*tests with Welch correction for unequal variance, paired *t* tests, or repeated-measures ANOVA with Sidak multiple comparisons test, as appropriate. Probability distributions were compared using the Kolmogorov-Smirnov test. Concentration-response data was modeled using a 4-parameter dose-response with the minima of the curves constrained to zero. Calculated curve-fit parameters (half-maximal effective concentration; EC_50_) are presented with 95% CIs and were compared statistically using the extra sum-of-square *F* test. As this was an exploratory study, an a priori power analysis was not conducted. All statistical analysis was performed using GraphPad Prism Version 6 (GraphPad Software, La Jolla, CA). A *P* value of <0.05 was considered statistically significant.

## Results

### Phenylephrine-Induced Contraction Is Enhanced in Hypertension

Contraction in small mesenteric arteries was investigated in en face arteries (Figure S1 and S2 and Movie S1, 156±7 µm passive diameter for SHR, 163±8 µm passive diameter for WKY, n=5 for each group). A depolarizing KCl (70 mmol/L) bath solution induced rapid contractions that plateaued within 1 minute (Figure S3A). The magnitude of contraction induced by KCl did not differ between strains (Figure S3B; 38±3 % of initial diameter for SHR, 45±3 % of maximum for WKY; n=4), though the maximal rate of contraction was higher in arteries from normotensive rats (Figure S3B; 2.2±0.1 % s^-1^ for SHR, 3.6±0.2 % s^-1^ for WKY; n=4). On restoration of normal KCl (4.7 mmol/L), arteries relaxed back to resting levels. Neither the magnitude nor the rate, of this relaxation, was different among strains (Figure S3C; 86±3 % relaxation for SHR, 73±12 % relaxation for WKY; rate of 0.5±0.1 %·s^-1^ for SHR, 0.7±0.1·% s^-1^ for WKY; n=4).

In the next series of experiments, vasoconstriction was induced using the selective α_1_-adrenergic receptor agonist, phenylephrine (Figure 1). Phenylephrine produced concentration-dependent contractions (Figure 1A). The phenylephrine concentration-response relationship in SHR (EC_50_=1.4 μM; 95% CI, 0.8–2.5 μM; n=5) was shifted to the left when compared with that in WKY (EC_50_=3.0 μM; 95% CI, 2.9–3.2 μM; n=5). The contractile response to phenylephrine was significantly enhanced by a further increase in circumferential stretch of ≈10% in both WKY and SHR (Figure S4). At this increased level of stretch, arteries from SHR (EC_50_=0.9 μM; 95% CI, 0.7–2.2 μM; n=5) remained significantly more sensitive to phenylephrine than those from WKY (EC_50_=2.0 μM; 95% CI, 1.9–2.1 μM; n=5). These data demonstrate that alpha-adrenergic receptor–mediated vasoconstriction is enhanced in hypertension.

### Endothelial, Ca^2+^-Dependent Negative Feedback Is Impaired in Hypertension

We have previously shown that the sensitivity of small mesenteric arteries to phenylephrine is enhanced after removal of the endothelium or blockade of endothelial IP_3_-mediated Ca^2+^ signaling.^43^ To determine if endothelial Ca^2+^-dependent modulation of vasoconstrictor responses is disrupted in hypertension, we simultaneously measured vascular reactivity and endothelial Ca^2+^ levels before and after buffering endothelial Ca^2+^ using the chelator, BATPA-AM (Figure 2 and Figure S2). As with the cumulative concentration-response relationships, contractions to a submaximal concentration of phenylephrine (1 µmol/L) were greater in en face arteries from hypertensive animals than in those from normotensive controls (Figure 2A and 2B; 9±2 % of initial diameter for SHR, 19±4 % of initial diameter for WKY; n=7 in each group). Acetylcholine (1 µmol/L) induced robust vasodilations of the preconstricted arteries. The dilations were similar in each experimental group (Figure 2A and 2C, 79±4 % for SHR, 77±4 % for WKY; n=7 in each group). Higher concentrations of acetylcholine (10 µmol/L) also evoked vasodilation of contracted arteries but did not evoke contraction of quiescent arteries (Figure S5), suggesting that acetylcholine does not cause the release of endothelium-dependent contractile factors in this model.

Buffering endothelial Ca^2+^ with BAPTA significantly potentiated contractile responses to phenylephrine in WKY (≈4-fold) and SHR (≈3-fold), such that contractions in each strain were similar after BAPTA treatment (Figure 2B; 40±7 % of initial diameter for SHR, 41±3 % of initial diameter for WKY; n=5 in each group). The potentiation, rather than attenuation, of phenylephrine-induced contraction demonstrates that smooth muscle cells were not significantly loaded with BAPTA. In contrast to its effect on contraction, BAPTA attenuated vasodilator responses to acetylcholine (Figure 2C; 22±7 % for SHR, 10±4 % for WKY; n=5 in each group). These data indicate that endothelial Ca^2+^-dependent processes limit contraction to a greater extent in normotensive (WKY) controls than in hypertensive (SHR) animals.

Because endothelial Ca^2+^-dependent processes normally limit contraction by activating vasodilator pathways, we expected endothelial Ca^2+^ levels to be higher in WKY than SHR endothelium. However, resting endothelial Ca^2+^ levels were significantly higher in SHR than WKY endothelium (Figure 2D; 1765±193 arbitrary fluorescence unit for SHR, 1159±100 AFU for WKY; n=5 in each group). BAPTA significantly reduced resting endothelial Ca^2+^ levels in WKY and SHR animals (Figure 2D; 1154±146 AFU for SHR, 795±77 AFU for WKY; n=5 in each group). Acetylcholine-evoked increases in endothelial Ca^2+^ levels (measured before the onset of relaxation) of phenylephrine-constricted vessels were similar in SHR and WKY (Figure 2E, 0.52±0.09 ΔF/F_0_ for SHR, 0.33±0.08 ΔF/F_0_ for WKY; n=5 in each group) and were prevented by BAPTA (Figure 2E; −0.01±0.01 ΔF/F_0_ for SHR, 0.01±0.01 ΔF/F_0_ for WKY; n=5 in each group).

Taken together, the results thus far suggest that (1) Ca^2+^ activity in the endothelium inhibits vascular smooth muscle cell contraction; (2) the endothelial Ca^2+^-dependent inhibition of vascular tone is significantly reduced in hypertension; and (3) the reduced ability of the endothelium to oppose contraction in hypertension is not explained by global endothelial Ca^2+^ levels.

### Local Endothelial Ca^2+^ Signaling Is Impaired in Hypertension

**Figure 3.**
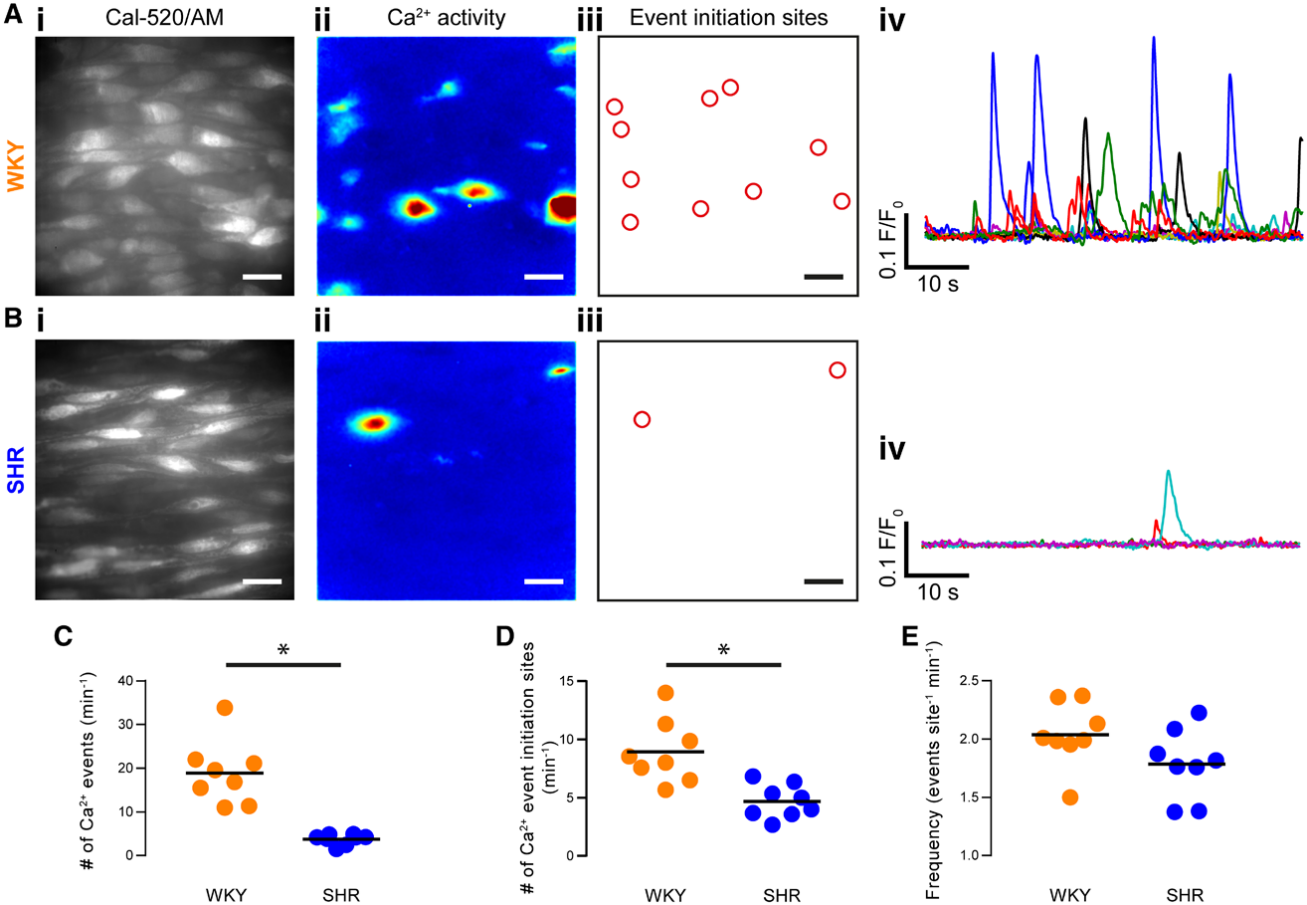
Spontaneous, local endothelial Ca^2+^ signaling is impaired in hypertension. **A** and **B**, Endothelial cells in intact arteries from normotensive rats (Wistar Kyoto [WKY], **A**) and spontaneously hypertensive rats (SHR; **B**) exhibit local subcellular Ca^2+^ signals under basal conditions (ie, in the absence of either mechanical or pharmacological stimulation). Scale bars=20 µm. **i**, High-resolution and wide field-of-view (×100 objective, NA=1.3, ≈13.3 × ≈13.3 µm field, ≈50 whole or partial cells) gray-scale images (**left**) of the endothelium of intact arteries (≈50 cells) loaded with the Ca^2+^ indicator, Cal-520-AM; (**ii**) composite images showing the SD of intensity from 1-min recordings of Ca^2+^ activity of the corresponding field of endothelium shown in **i**; (**iii**) automatically generated regions of interest placed at the center of Ca^2+^ event initiation sites, from the same data illustrated in **i** and **ii**; and (**iv**) baseline-corrected Ca^2+^ signals (F/F_0_) extracted from the ROIs shown in **iii**. **C** through **E**, Summary data showing: (**C**) the number of event initiation sites; (**D**) the total number of Ca^2+^ events; and (**E**) the average event frequency at each active event initiation site. WKY is denoted by orange data points and SHR by blue data points. Each data point illustrates the mean value obtained from at least 3 separate fields of endothelial cells from a single animal, the black line indicates the mean of means (n=8 animals for both WKY and SHR). *P<0.05, unpaired *t* test with Welch correction.

Under basal conditions, endothelial cells exhibit spontaneous, subcellular (local) Ca^2+^ signals.^23,44^ These local signals modulate vasodilator pathways but may be missed by global analyses.^45^ We hypothesized that hypercontractile vessel responses in hypertension arise from impaired local basal endothelial Ca^2+^ activity. To test this hypothesis, we examined basal endothelial Ca^2+^ dynamics at high temporal (20 Hz) and spatial (×100 magnification, NA-1.3; 130 nm projected pixel size; ≈133 µm × 133 µm field-of-view; Figure 3) resolution. Ca^2+^ event initiation sites were identified automatically using algorithms obtained from a well-established tool (FLIKA) for automated analysis of localized events in Ca^2+^ imaging data,^37^ which was incorporated into our own Ca^2+^ signal analysis software suite.^25^ This analysis provided reliable quantification of local Ca^2+^ signals and outperformed manual visual inspection of the data (Figure S6).

WKY and SHR endothelial cells exhibited substantial local Ca^2+^ activity under basal conditions (Figure 3A and Movies S2 and S3). These local signals arise from IP_3_R-mediated release of Ca^2+^ from the internal store (they persist in Ca^2+^-free extracellular bathing solution and are blocked by 2-Aminoethoxydiphenyl borate and cyclopiazonic acid).^46^ There were significantly fewer basal Ca^2+^ events in SHR than in WKY endothelia (Figure 3A through 3C; 3.8±0.4 events/field for SHR, 18.9±2.5 events/field for WKY; n=8 in each group). There was also a significantly lower number of sites giving rise to spontaneous Ca^2+^ activity in hypertension compared to normotensive controls (Figure 3D; 4.7±0.5 sites/field for SHR, 8.9±1.0 sites/field for WKY; n=8 in each group; *P*<0.05; unpaired *t* test with Welch correction). However, within active initiation sites, the average event firing rate (frequency) was similar in each experimental group (Figure 3E; 1.8±0.1 events/site for SHR, 2.0±0.1 events/site for WKY; n=8 in each group; *P*=0.1; unpaired *t* test with Welch correction). These results suggest that, in hypertension, although endothelial cells remain able to encode information within the frequency of Ca^2+^ signals, the collective activity across the endothelial network is impaired.

Because vascular reactivity to phenylephrine is increased after buffering endothelial Ca^2+^ (Figure 2), we hypothesized that the increased contractile responses to phenylephrine at higher circumferential stretch (Figure S4) might arise from impaired basal endothelial Ca^2+^ signaling. We assessed basal Ca^2+^ activity before and after a ≈10% increase in circumferential stretch. In support of our hypothesis, stretch reduced Ca^2+^ activity in the endothelium of both WKY and SHR (Figure S7).

**Figure 4.**
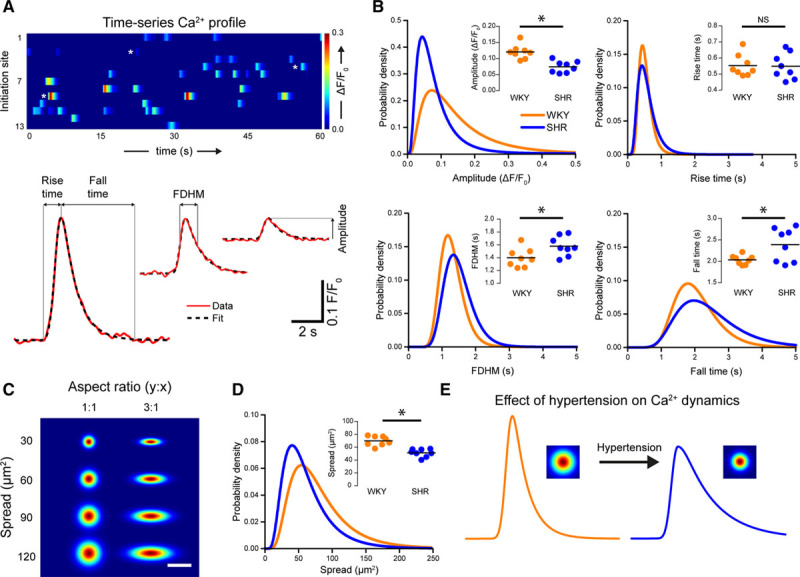
Ca^2+^ signaling dynamics are altered in hypertension. **A**, Basal Ca^2+^ signals from a Wistar Kyoto (WKY) control displayed as parallel heat maps (**top**) and Ca^2+^ transients (**bottom**; red line) shown with exponentially modified gaussian fits. The Ca^2+^ event traces are indicated by heat map by white stars. The model was used to extract the indicated temporal parameters. **B**, Probability distributions and summary data (insets) of amplitude, full duration at half maximum (FDHM), rise time, and fall time for WKY (orange) and spontaneously hypertensive rat (SHR; blue) animals. Distributions are log-normal curves fitted to pooled data (N=753 events from 8 animals for WKY; N=314 events from 8 animals for SHR). Because of the log-normal distribution, a logarithmic transform was applied to raw data. Summary data show geometric means (calculated by back transforming the mean of log-transformed data). *Significance (*P*<0.05) using unpaired *t* tests with Welch correction on the log-transformed data. **C**, Example plots of 2-dimensional gaussians demonstrating the function used to calculate spatial spread. Functions increase in area down the vertical, and the 2 columns illustrate the effect of alterations in aspect ratio. Spread was determined by calculating the elliptical area covered by the 2-dimensional function. Scale bar=20 µm. **D**, Probability distribution and summary data (inset) showing the effect of hypertension on the spread of Ca^2+^ events from their point of origin. Summary data show geometric means of data obtained from individual animals. *Significance (*P*<0.05) using unpaired *t* test with Welch correction on the log-transformed data (n=8). **E**, Illustration of the effect of hypertension on basal endothelial Ca^2+^ signals. In hypertension, Ca^2+^ signals are smaller in amplitude and spread but persist for longer due to an increase in the time taken for the Ca^2+^ level to return to baseline. NS indicates nonsignificant.

Many cells encode information in the amplitude, duration (FDHM), rise time, fall time, and spread of a Ca^2+^ transient to target particular Ca^2+^-dependent effectors.^47^ To determine if these signaling metrics are altered in hypertension, we plotted frequency distributions of each parameter by pooling data from all experiments (Figure 4). Temporal features were extracted from a Gaussian-modified exponential function that was fit to each Ca^2+^ event (Figure 4A). The spread of Ca^2+^ from each initiation site during each event was obtained by fitting a 2-dimensional gaussian function to *z*-projections of the Ca^2+^ signal (SD of pixel intensity; Figure 4C). The distributions of each parameter (amplitude, FDHM, rise time, fall time, and spatial spread) were log-normal in WKY and SHR, suggesting multiplicative (rather than additive) variability in the response. Such distributions are consistent with an amplification process in which released Ca^2+^ recruits additional channels.^48,49^

The distributions of Ca^2+^ event amplitudes (Figure 4B, top left), FDHM (Figure 4B, bottom left), fall times (Figure 4B, bottom right) and spatial spreads (Figure 4D) differed significantly in WKY and SHR rats (*P*<0.05, 2-sample Kolgorov-Smirnov tests). No difference was detected in the distributions of event rise times (Figure 4B, top right; *P*=0.47; 2-sample Kolmogorov-Smirnov test). An analysis of means (Figure 4B and 4D, insets) revealed that the amplitude and spatial spread of Ca^2+^ signals were significantly lower in SHR (0.07 ΔF/F_0_, 95% CI, 0.06–0.09 ΔF/F_0_ for amplitude; 50.9 s, 95% CI, 45.8–56.5 s for spread), compared with WKY (0.12 ΔF/F_0_, 95% CI, 0.10–0.14 ΔF/F_0_ for amplitude; 69.7 s, 95% CI, 63.5–76.2 s for spread). In contrast, the FDHM and fall time were both significantly higher in SHR (1.57 s, 95% CI, 1.45–1.70 s for FDHM; 2.4 s, 95% CI, 2.1–2.7 s for fall time), compared with WKY (1.39 s, 95% CI, 1.28–1.52 s for FDHM; 2.0 s, 95% CI, 1.9–2.1 s for fall time) animals. No difference in the mean rise time was detected between groups (0.54 s, 95% CI, 0.48–0.61 s for SHR; 0.55 s, 95% CI, 0.50–0.61 s for WKY; n=8).

Collectively, these data demonstrate that basal subcellular Ca^2+^ signals occur less often and are smaller in amplitude and spatial spread, but last longer in hypertension than in normotensive controls (Figure 4E). To investigate if the decrease in basal Ca^2+^ activity observed in hypertension arose from alterations in Ca^2+^ store content, we examined Ca^2+^ responses induced by the ionophore, ionomycin in a Ca^2+^ free physiological saline solution. Total store content (assessed by the area-under-the-curve of ionomycin-evoked global Ca^2+^ signals) was similar in WKY and SHR (Figure S8A, n=6 in each group). We next investigated if there were any difference in the ability of IP_3_ to evoke Ca^2+^ release. To do this, we bypassed phospholipase C-dependent IP_3_ production by photolyzing a caged photolabile version of the inositide. In the absence of external Ca^2+^, there were no significant differences in the amplitude of IP_3_-mediated endothelial Ca^2+^ responses of WKY and SHR (Figure S8B, n=8 in each group), suggesting that the ability of IP_3_ to evoke a Ca^2+^ response is unaltered in hypertension. To assess if Ca^2+^ removal mechanisms were altered in hypertension, we calculated the maximum rate of Ca^2+^ removal after photolysis of caged IP_3_. The maximum rate of decline of the caged IP_3_-evoked Ca^2+^ response was similar in WKY and SHR (Figure S8C, n=8 in each group).

Collectively, these data demonstrate that basal subcellular Ca^2+^ signals occur less often, are smaller in amplitude and spatial spread, but last longer in hypertension than in normotensive controls (Figure 4I). The cause of dysfunctional spontaneous Ca^2+^ activity is not resolved but is unlikely to be because of alterations in-store content, the ability of IP_3_ to evoke Ca^2+^ release, or Ca^2+^ removal via either sarco/endoplasmic reticulum Ca^2+^-ATPase, plasma membrane Ca^2+^ pump, or Na^+^-Ca^2+^ exchanger.

### IEL Hole Distribution Is Altered in Hypertension

**Figure 5.**
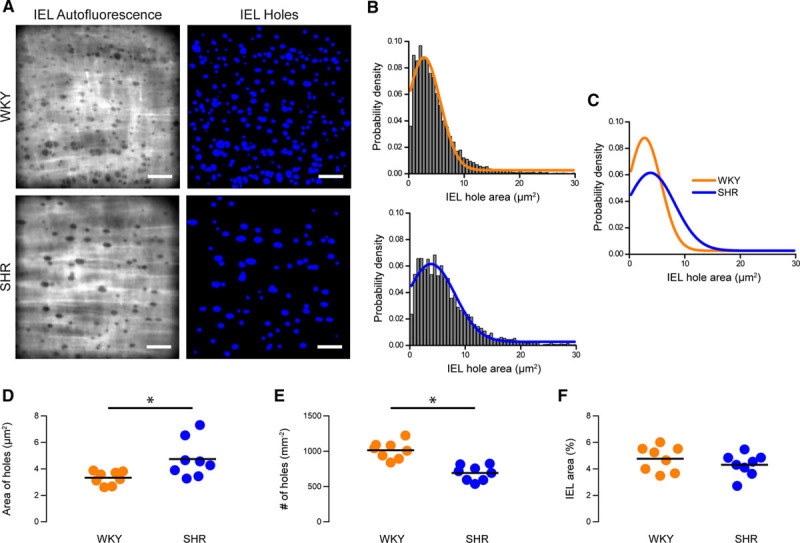
Structural internal elastic lamina (IEL) alterations in hypertension. **A**, Representative images of IEL holes in mesenteric arteries from Wistar Kyoto (WKY) control (**top** row) and hypertensive (**bottom** row) animals. **Left**, Raw elastin autofluorescence images. These images were processed and inverted to highlight IEL holes (**right**). Scale bars=20 µm. **B** and **C**, Probability density distributions of IEL hole size in mesenteric arteries from WKY (**B**, **top**; orange) and spontaneously hypertensive rat (SHR; **C**, **top**; blue) animals showing the effect of hypertension on the spread of Ca^2+^ events from their point of origin (**C**). Distributions are log-normal curves fitted to pooled data (N=8320 IEL holes from 8 animals for WKY; N=4368 holes from 8 animals for SHR). **D**–**F**, Summary data showing the effect of hypertension on IEL hole area (**D**), IEL hole density (**E**), and the area of IEL occupied by holes (**F**). In **D**, geometric means (calculated by back transforming the mean of log-transformed data) of data from individual animals is shown. In **G** and **H**, means of untransformed data are shown. *Significance (*P*<0.05) using unpaired *t* tests with Welch correction on log-transformed (**D**) or untransformed (**E** and **F**) data.

Endothelial cells are separated from smooth muscle cells by the IEL. Gaps in the elastic lamina (IEL holes) provide sites that permit signaling to occur between endothelial cells and smooth muscle cells. Signals may spread directly between the two cell types, or IEL holes may provide routes for the diffusion of vasoactive factors.^18,19,50^ IEL holes may be identified by a lack of signal when the IEL is visualized using elastin autofluorescence^19^ or with the aid of fluorescence indicators.^51^ To investigate the possibility of alterations in the extent of endothelial-smooth muscle cell coupling via the IEL in hypertensive animals, we visualized the autofluorescence emission of the IEL upon excitation with near-UV (390 nm) light (Figure 5A).

There was an increase in the proportion of larger IEL holes in hypertension when compared with WKY controls (Figure 5B through 5C; *P*<0.05, 2-sample Kolmogorov-Smirnov tests). Mean IEL hole area was also greater in hypertensive SHR than normotensive WKY controls (Figure 5D; 4.6 µm^2^, 95% CI, 3.6–5.8 µm^2^ for SHR; 3.3 µm^2^, 95% CI, 2.9–3.8 µm^2^ for WKY; n=8). However, although total IEL hole area was higher, the density of IEL holes was lower in SHR compared with WKY (Figure 5E; 691±38 holes/mm^2^ for SHR, 1015±45 holes/mm^2^ for WKY; n=8 in each group). Thus, IEL holes are fewer in number but larger in size in hypertensive animals. No difference was detected in the mean percentage area of IEL occupied by holes in each group (Figure 5F; 4.3±0.3 for SHR, 4.8±0.3 for WKY; n=8 in each group). Previous studies on chemically fixed large (carotid) arteries have also suggested that the number of holes in the internal elastic lamina was altered in hypertension; the area occupied by fenestrations was reduced in carotid arteries.^52,53^ These results raise the possibility that endothelial-smooth muscle cell coupling may be compromised in hypertension.

### Endothelial Ca^2+^ Events Are Decoupled From MEPs in Hypertension

**Figure 6.**
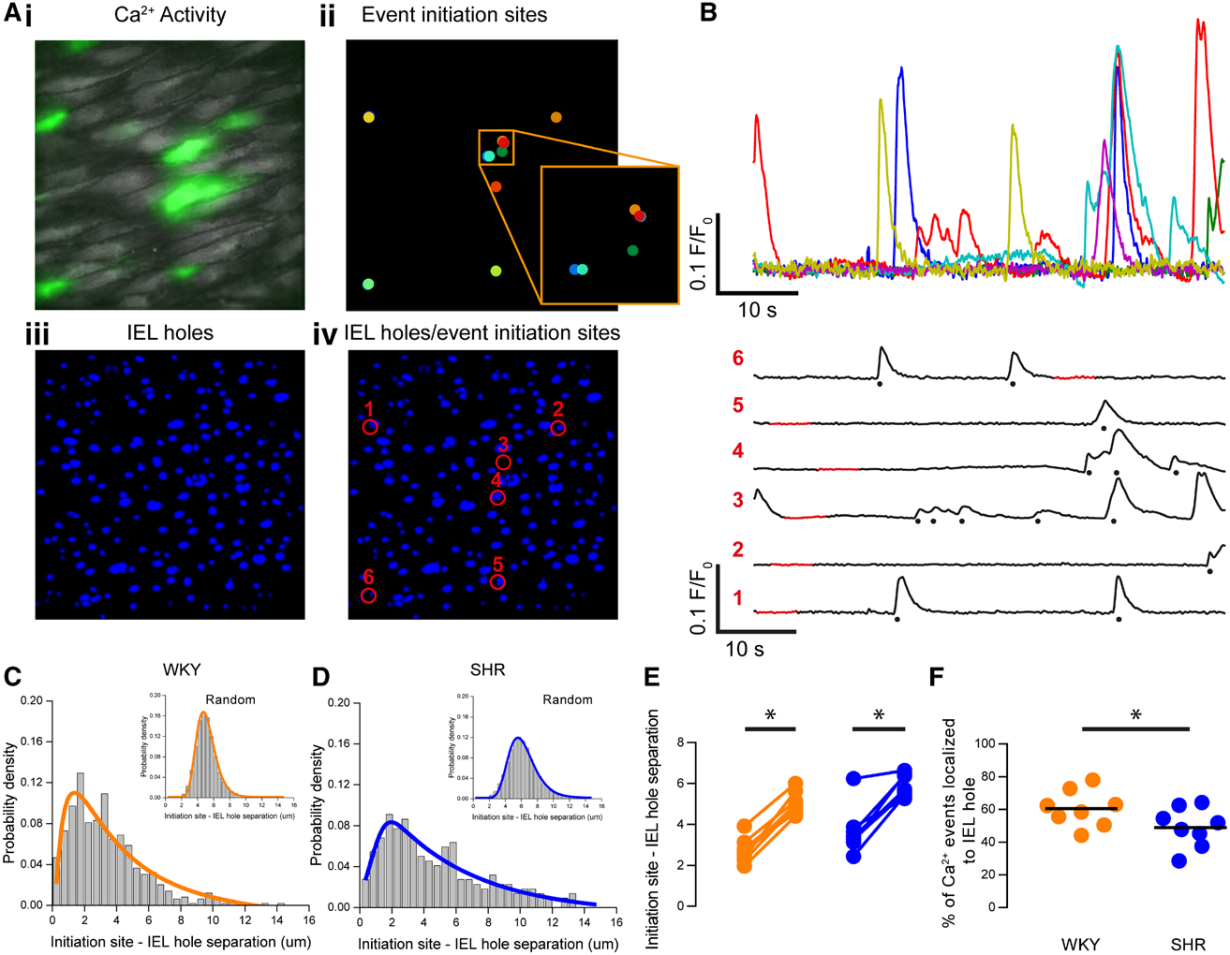
Decoupling of Ca^2+^ events from myoendothelial junctions in hypertension. **A**, Composite image showing endothelial Ca^2+^ activity in green (**i**), Ca^2+^ event initiation sites (**ii**), internal elastic lamina (IEL) holes (**iii**), and an overlay of Ca^2+^ event initiation sites and IEL holes (**iv**). **B** and **C**, Ca^2+^ signals extracted from the sites shown in **Aiv**. In **C**, the Ca^2+^ signals have been spread out for clarity. The red portion of the signal demarcates the automatically determined baseline region, and detected events are indicated by stars. **D** and **E**, Probability density distributions illustrating the log-normal distribution of the measured separation between Ca^2+^ event initiation sites and IEL holes for Wistar Kyoto (WKY; **D**) and spontaneously hypertensive rat (SHR; **E**). Distributions arising from randomly permuted data are also shown (insets). **F**, Paired summary data showing that the mean centroid-centroid distance between Ca^2+^ event initiation sites and the nearest IEL observed was significantly lower than expected than a randomized distribution Ca^2+^ event initiation sites in both SHR and WKY endothelium (paired data points). **G**, Summary data showing a reduction in the percentage of Ca^2+^ events localized to IEL holes in hypertension. *Significance (*P*<0.05) using paired *t* tests on log-transformed data (**F**) or unpaired *t* test with Welch correction (**G**).

To explore the possibility of decreased signaling at MEPs in hypertension, we investigated the colocalization of Ca^2+^ signals with holes in the IEL. In previous studies, investigators have inferred colocalization of Ca^2+^ events and IEL holes by eye.^41,54^ Other studies have used a more quantitative approach by using a distance threshold (eg, 5 µm) to indicate colocalization.^20,55,56^ In line with these observations, in the present study, Ca^2+^ events did appear to occur in the vicinity of IEL holes (Figure 6A and 6B). To examine Ca^2+^ event-IEL hole coupling, we compared the extent of overlap between Ca^2+^ signal initiation sites and IEL holes with that expected from a completely random distribution.^57^ For both WKY and SHR, the distributions of the distance between each Ca^2+^ event and corresponding nearest IEL hole in the randomized data were significantly different from the measured data (Figures 6C and 6D; 2-sample Kolmogorov-Smirnov tests). An analysis of means confirmed that Ca^2+^ events occurred nearer to IEL holes than would be expected if the Ca^2+^ events initiated randomly throughout the cytoplasm for both WKY (2.7 µm, 95% CI, 2.2–3.2 µm for observed; 5.0 µm^2^, 95% CI, 4.6–5.5 µm for random; n=8) rats and SHR (3.5 µm, 95% CI, 2.8–4.4 µm for observed; 5.8 µm, 95% CI, 5.4–6.3 µm for random; n=8; Figure 6E). This result suggests that local Ca^2+^ signaling events predominate near IEL holes.

The extent to which a local Ca^2+^ event is coupled to an IEL hole influences how likely it is to regulate smooth muscle contraction. We hypothesized that coupling of local Ca^2+^ signals to IEL holes would be disrupted in hypertension. To test this hypothesis, we determined the fifth percentile of initiation site-IEL hole separation for each random dataset and considered a Ca^2+^ event to be coupled to an IEL hole if it occurred within this distance. The percentage of Ca^2+^ events localized to IEL holes was significantly lower in hypertensive (SHR, 49±4 %) when compared with normotensive (WKY, 61±4 %) animals (Figure 6F; n=8 for each group). This data suggests that hypertension is associated with reduced coupling of local Ca^2+^ signals to smooth muscle cells via the MEP.

## Discussion

Intracellular Ca^2+^ signaling is central to endothelial control of cardiovascular activity. Endothelial Ca^2+^ signals control the synthesis and release of macromolecules involved in angiogenesis,^58,59^ inflammatory responses,^60,61^ and the regulation of vascular smooth muscle contraction (reviewed^62^). In the endothelium, sites that make contact with smooth muscle cells (MEPs), have emerged as crucial Ca^2+^ signaling microdomains that are pivotal in the regulation of vascular function. These sites are enriched with Ca^2+^-dependent effector proteins that modulate the contractile state of smooth muscle.^18,19,50^ Our results demonstrate that basal, local IP_3_-mediated endothelial Ca^2+^ signals occur less frequently and are lower in amplitude in hypertension when compared with normotensive controls. These local Ca^2+^ signals occur predominantly at contact sites with smooth muscle cells (MEPs) but are decoupled from MEPs in hypertension. The deficiencies in IP_3_-mediated Ca^2+^ signaling reduce the ability of the endothelium to oppose vascular tone and can be mimicked in normotension via selective buffering of endothelial Ca^2+^. These results demonstrate that changes in local, IP_3_-mediated, Ca^2+^ signaling at MEPs contribute to the impaired endothelial control of vascular tone and the increased contractile state of smooth muscle in hypertension.

Various proposals exist to explain the impaired endothelial-dependent control of blood vessel tone in hypertension. The proposals include alterations in connexons and NADPH oxidase expression and altered Ca^2+^-activated K^+^ channel activity.^63–68^ The changes decrease endothelium-dependent hyperpolarization and NO production,^63–67,69^ each of which are Ca^2+^-dependent processes. However, surprisingly little is known about endothelial Ca^2+^ signaling in hypertension, and the few existing reports are contradictory. For example, impaired responses to bradykinin and endothelin-1 have been observed in cultured aortic endothelial cells derived from hypertensive rats.^9,17^ In contrast, the endothelial Ca^2+^ response to muscarinic receptor activation is increased in native aortic endothelial cells from hypertensive rats (SHR),^16^ or unaltered in native murine mesenteric artery endothelial cells (angiotensin II–induced hypertension).^20^

A possible explanation for the discrepancies may lie in the subtlety of various Ca^2+^ signaling modalities. In unstimulated endothelial cells in situ^23^ and in vivo,^51^ local IP_3_-mediated Ca^2+^ events occur at MEPs where they exert a persistent anticontractile influence by activating Ca^2+^-sensitive potassium channels and eNOS.^23,24,63,70^ These local events are termed Ca^2+^ pulsars and are reportedly distinct from the elementary Ca^2+^ blips and puffs that give rise to Ca^2+^ waves in Xenopus oocytes.^65,66^ Another local Ca^2+^ signaling modality occurs via Ca^2+^ influx through transient receptor potential channels (eg, TRPV4^56^ or TRPA1^71,72^ sparklets). Like pulsars, transient receptor potential channel-mediated influx events also couple to MEPs where they activate Ca^2+^ activated potassium channels and NO synthesis directly^20,56,73^ or indirectly via Ca^2+^-induced Ca^2+^ release from the endoplasmic reticulum.^28,43,74^ As transient receptor potential channel-mediated sparklets occur very infrequently under basal conditions,^20,56^ they do not seem to contribute to the innate ability of the endothelium to oppose vasoconstriction. Instead, a reduction in localized TRPV4-mediated Ca^2+^ influx events underlies a loss of muscarinic receptor–mediated vasodilation in a murine model of angiotensin II–induced hypertension.^20^ This observation demonstrates that local Ca^2+^ signaling pathways contribute to vascular dysfunction in hypertension. However, despite evidence pointing to a critical role in controlling vascular tone, local IP_3_-mediated Ca^2+^ signaling in hypertension has not been previously investigated.

Consistent with our previous results in another strain of rat,^46^ mesenteric endothelial cells exhibit spontaneous IP_3_-mediated Ca^2+^ release events in both WKY and SHR. The events had continuous distributions of amplitude, duration, and spread and ranged from highly localized release events to larger, but still subcellular, Ca^2+^ waves. The continuum of release events presumably arises from variations in the recruitment of neighboring IP_3_Rs. The results suggest that IP_3_-mediated Ca^2+^ signaling in rat endothelium is an analog process (see also Burdyga et al^75^), unlike the digital process (recruitment of additional sites, increase in frequency) described for Ca^2+^ pulsars in murine mesenteric arteries. However, despite differences in the kinetic profiles of Ca^2+^ events in murine and rat endothelium, both signaling modalities predominantly originate at or near MEPs so are well placed to contribute to the regulation of vascular tone by endothelium-derived hyperpolarization and NO production.

Basal IP_3_-mediated endothelial Ca^2+^ signals occurred less frequently, were smaller in amplitude and more spatially confined, but lasted longer (ie, had a slower rate of decline) in hypertension. Local Ca^2+^ signals were also positioned further from IEL holes in SHR than in WKY controls, suggesting that the normal coupling of local Ca^2+^ signals and MEPs is disrupted in hypertension. These alterations may decrease the occurrence of endothelium-dependent hyperpolarization and NO production at the MEPs and, as a result, increase vascular reactivity to contractile agents.^63–67,69^ In support of this conclusion, selectively buffering endothelial Ca^2+^ signals using the chelator, BAPTA, increased contractions to phenylephrine and abolished the difference in sensitivity of arteries from normotensive and hypertensive animals to the contractile activator.

Our observations of an impaired inhibitory role of the endothelium in hypertension are in line with several previous reports.^76–81^ Reduced endothelium-dependent vasodilation or increased endothelium-dependent contraction are reported frequently in hypertension. For example, in precontracted aorta, acetylcholine-evoked endothelium-dependent dilation may be blunted in hypertensive rats (SHR) when compared with normotensive (WKY) controls.^82–85^ In quiescent aorta^50,51^ and mesenteric arteries^86,87^ (ie, in the absence of any contractile agent) of SHR, but not WKY, high concentrations (>1 µmol/L) of acetylcholine may evoke cyclooxygenase-sensitive, endothelium-dependent contractions^84,85^ that are enhanced after inhibition of NO synthesis.^85,88^ These observations are interpreted together to suggest that impaired endothelial control of smooth muscle cell function in hypertension arises from an imbalance in the complex interplay of endothelium-derived relaxing and contracting factors.^89^ Notwithstanding, reduced dilation is not universally observed in hypertension.^76,90,91^ Neither are endothelium-dependent contractions to acetylcholine.^92,93^ In the present study, although contractile responses of mesenteric arteries were augmented, vasodilation was unaltered in hypertension, and we did not observe acetylcholine-induced contraction in either WKY or SHR (Figure S5). Thus, alterations in agonist-evoked relaxation or the release of endothelium-dependent contractile factors is unlikely to explain the hypercontractility observed in the present study. Differences in the precise manifestation of endothelial dysfunction and the extent of impaired vascular reactivity likely arises from the degree of hypertension and the age of animals under study.^76,78,92,94–96^

As the ability of the endothelium to oppose constriction is dependent on the nature of the vasoconstrictor stimulus,^79,97^ it may be that the endothelial IP_3_-mediated inhibitory pathway described here for adrenoceptor-mediated contractions may also modulate pressure-induced constriction. Endothelial Ca^2+^ activity is suppressed at high intraluminal pressure and inversely correlates with the development of myogenic tone.^54^ Thus, although our experiments did not address mechanisms of pressure-induced constriction, the enhanced myogenic tone observed in the SHR^98^ would be anticipated from the impaired endothelial Ca^2+^ activity observed in SHR. Indeed, contractile responses to phenylephrine and basal Ca^2+^ activity were negatively correlated with the level of stretch applied to arteries. In addition, although not universally observed,^99^ adrenoceptor agonists may activate endothelial cells directly^100–103^ or indirectly^54,70,104–108^ to amplify basal MEP-associated endothelial Ca^2+^ activity and further limit vasoconstriction. In the present study, it was not possible to reliably assess whether or not adrenoceptor activation increased endothelial cell Ca^2+^ levels because of the smooth muscle contraction evoked by the adrenoceptor agonist.

The question arises as to why basal Ca^2+^ signaling is impaired and uncoupled from MEPs in hypertension. Though the spatiotemporal impairment (decreased frequency, reduced amplitude and spatial spread, slower rate of decline) is consistent with a reduction in the ability of SERCA to sequester Ca^2+^ into the endoplasmic reticulum,^67,68^ we did not find evidence of altered Ca^2+^ removal following Ca^2+^ release evoked by photolysis of caged IP_3_. In addition, the present results show the endoplasmic reticulum content was unchanged in hypertension. Rather than decreased Ca^2+^ uptake, impaired basal endothelial Ca^2+^ signaling may be due to reduced basal production of IP_3_ in the endothelium of hypertensive rats.

Uncoupling of IP_3_-mediated Ca^2+^ events from MEPs may be the result of a reduction in the expression of tethering proteins, as has been shown for the PKC (protein kinase C)-anchoring protein, A-Kinase Anchoring Protein 150, which couples TRPV4 Ca^2+^ influx channels to MEPs.^20^ However, the decrease in targeting of local Ca^2+^ signals to MEPs may also arise from the arterial remodeling that accompanies hypertension.^109–113^ In support of this scenario, we observed fewer IEL holes in hypertension, which likely corresponds to a lower number of MEPs and a decreased probability of an interaction between Ca^2+^ signals and MEPs. This proposal, if correct, would suggest that the change in Ca^2+^ signaling events that initiate at MEPs is a consequence rather than a cause of hypertension.

In conclusion, we show that basal IP_3_-mediated Ca^2+^ signaling is disrupted in the endothelium of arteries from hypertensive rats (SHR). In hypertension, local endothelial Ca^2+^ signals occur less often, are reduced in amplitude and spread, and are uncoupled from myoendothelial gap junctions. These basal endothelial Ca^2+^ signals limit vascular contraction, but this control is compromised in hypertension. These results support the view that local Ca^2+^ signals are important for endothelial-dependent modulation of smooth muscle contractile function and suggest that impaired local IP_3_-mediated Ca^2+^ signals in endothelial cells have widespread global consequences on vascular smooth muscle function in hypertension.

## Perspective

Projections between endothelial and smooth muscle cells (MEPs) contain several Ca^2+^-dependent effector proteins and act as a functional hub where endothelial signaling pathways merge to promote relaxation of smooth muscle cells. In hypertension, there is impaired Ca^2+^-dependent, endothelial control of vascular tone resulting in increased contraction. We demonstrate two changes in IP_3_-mediated Ca^2+^ signals at MEPs that explain the impaired endothelial control of vascular smooth muscle contraction. First, there is a reduced liberation of Ca^2+^ from active Ca^2+^ release sites, which are positioned near MEPs. Second, there is decoupling of (an increased distance between) active Ca^2+^ release sites and MEPs. Together, these alterations to myoendothelial Ca^2+^ signaling dynamics decrease endothelial control of vascular smooth muscle contraction and explain the increased smooth muscle cell contractility that is characteristic of hypertension.

## Sources of Funding

This work was funded by the Wellcome Trust (202924/Z/16/Z; 204682/Z/16/Z) and the British Heart Foundation (PG/16/54/32230; PG16/82/32439), whose support is gratefully acknowledged. We thank Margaret MacDonald for her excellent technical support

## Disclosures

None.

## Supplementary Material

## Novelty and Significance

### What Is New?

- Local endothelial Ca^2+^ activity at junctions between endothelial and smooth muscle cells is dysregulated in hypertension.
- Basal endothelial Ca^2+^ signals are less frequent and smaller in amplitude in hypertensive animals than in normotensive controls.
- The spatial organization of subcellular endothelial Ca^2+^ signals is disrupted in hypertension.
- Impaired basal endothelial Ca^2+^ signaling underlies the increased sensitivity to contractile agents observed in the spontaneously hypertensive rat.

### What Is Relevant?

- Hypertension is associated with impaired endothelial function leading to increased vasoconstriction, but the mechanisms involved are unclear.
- The decrease in endothelial function that begins in hypertension, and progresses the disease, may lead to additional cardiovascular complications such as atherosclerosis.
- Junctions between endothelial and smooth muscle cells are critical to the control of vascular contractility. Our results provide a new mechanistic target for endothelial dysfunction in hypertension by demonstrating that local IP_3_(inositol trisphosphate)-evoked Ca^2+^ signals are disrupted at the junction between the endothelium and smooth muscle in hypertension.

### Summary

- Local endothelial Ca^2+^ signals at junctions between endothelial and smooth muscle cells normally limit contraction but this Ca^2+^ signaling circuit is disrupted in hypertension resulting in increased vascular reactivity.
