# Supplementary material for: Increased Vascular Contractility in Hypertension Results From Impaired Endothelial Calcium Signaling
Source: Hypertension. 2019 Sep 23;74(5):1200–14. doi: 10.1161/HYPERTENSIONAHA.119.13791 (PMC6791503; doi:10.1161/HYPERTENSIONAHA.119.13791)
Supplement: Supplementary file 1 [file hyp-74-1200-s001.docx]

**SUPPLEMENTARY INFORMATION**

**Increased vascular contractility in hypertension results from impaired endothelial calcium signaling**

Calum Wilson, Xun Zhang, Charlotte Buckley, Helen R Heathcote, Matthew D Lee & John G McCarron

Department of Strathclyde Institute of Pharmacy and Biomedical Sciences, University of Strathclyde, SIPBS Building, 161 Cathedral Street, Glasgow G4 0RE, UK.

Correspondence to c.wilson@strath.ac.uk 44 141 548 4976 or [john.mccarron@strath.ac.uk)](mailto:john.mccarron@strath.ac.uk) 44 141 548 4119.

**Supplementary Figures**


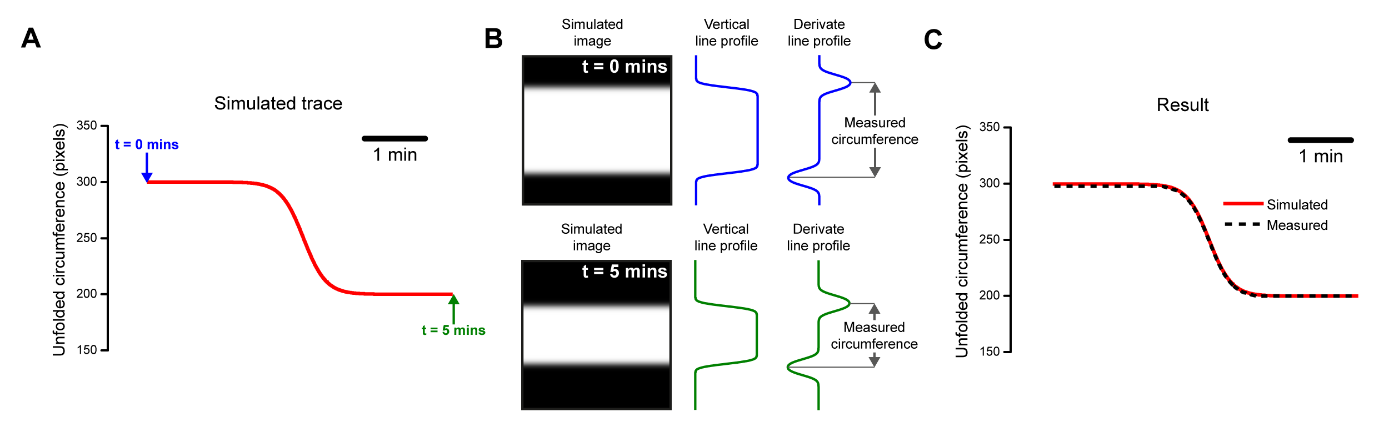


**Figure S1 – Automated quantification of arterial tone in *en face* artery preparations.** A) To confirm the tracking efficiency of the algorithm, synthetic test data was generated from a simulated trace (logistic function) of artery diameter (circumference/width). B) Overview of the algorithm used to extract artery dimensions. The position of the artery edges may be automatically extracted from the derivative (right) of line intensity profiles (middle) that bisect the longitudinal axis of the artery in *en face* imaging data (left panels; synthetic test data). The width of the preparation equates to the unfolded circumference of the intact artery. C) Comparison of output data (dashed black line) with original simulated trace (red line).


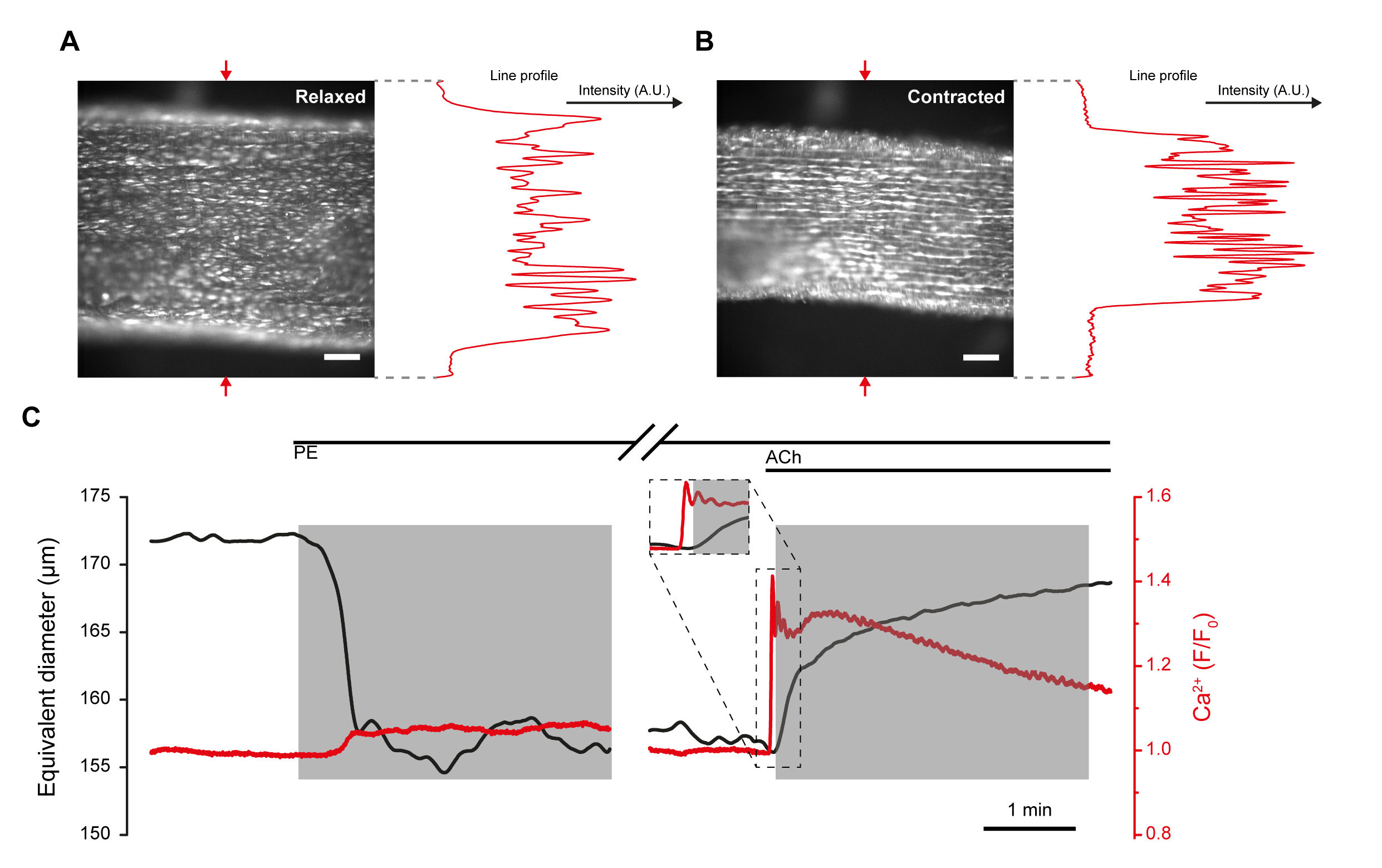


**Figure S2 – Simultaneous measurement of endothelial Ca^2+^ and artery tone in en face arteries.** A-B) Wide field (16X objective, NA = 1.3, ~832 × ~832 µm field) grey-scale images of the endothelium of an artery loaded with the Ca^2+^ indicator, Cal-520-AM before (A) and after (B) stimulation with stimulation of the smooth muscle with the alpha-adrenoceptor agonist, phenylephrine (1 µM). The red lines show line intensity profiles across the corresponding image (position indicated by red arrows), used to quantify smooth muscle cell contraction (see Supplementary Figure 1). Scale bar = 100 µm. C) A) Representative diameter (black) and global Ca^2+^ trace (red) from an artery stimulated with phenylephrine (PE, 1 µM) and then acetylcholine (ACh, 1 µM).


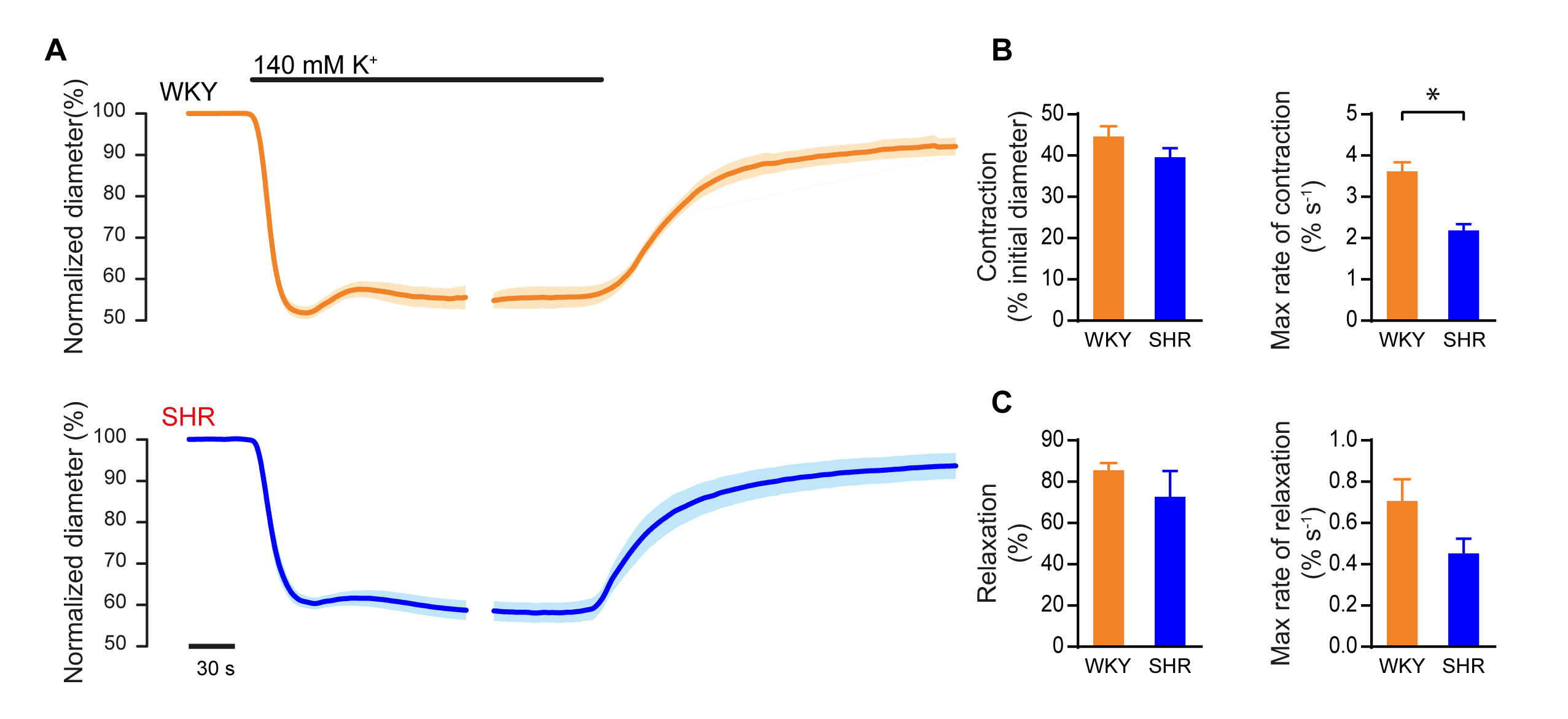


**Figure S3 – Contractions to potassium chloride are maintained in hypertension.** A) Representative artery diameter traces from normotensive (WKY, top) and hypertensive (SHR, bottom) animals in response to KCl (70 mM). B) Summary of KCl-induced response data illustrating the effects of hypertension on the magnitude (left) and rate (right) of the contraction. C) Summary of relaxation data illustrating the effects of hypertension on the magnitude (left) and rate (right) of the relaxation on wash out of KCl. Data are shown as mean values ± S.E.M (n = 4 for each). * indicates significance (p < 0.05) using unpaired t test with Welch’s correction.


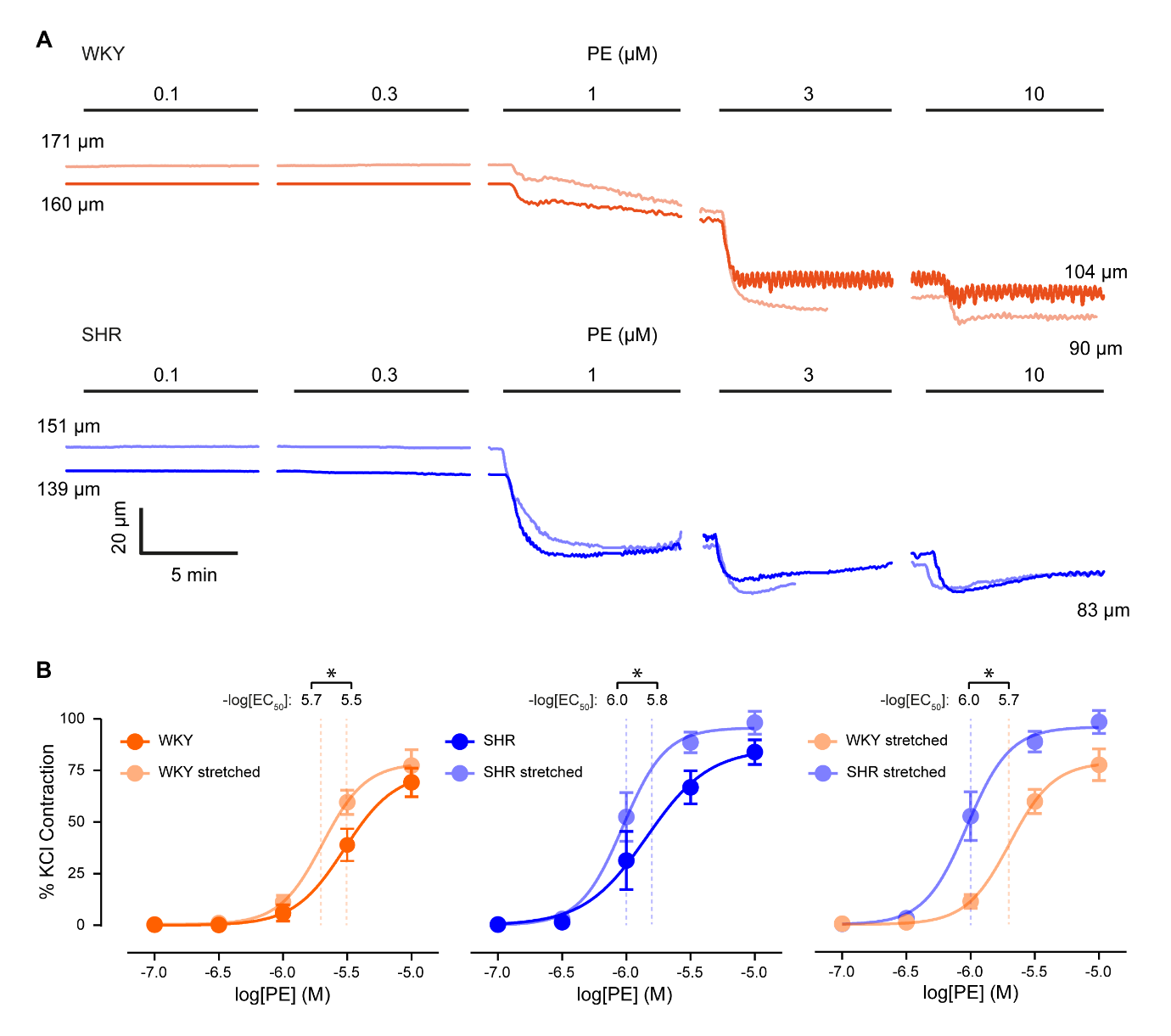


**Figure S4 – Circumferential stretch augments phenylephrine-induced contraction.** A) Representative artery diameter traces from normotensive (WKY, top) and hypertensive (SHR, bottom) animals in response to phenylephrine (PE). Traces show the mechanical response of a single artery from each strain before (bold line) and after (faint line) an increase in circumferential stretch. B) Concentration-response curves for the contractile effect of phenylephrine on small mesenteric arteries of WKY and SHR animals exposed to an increase in circumferential stretch. Contraction is expressed as a percentage of the maximum contraction induced by a depolarizing solution containing 140 mM K^+^ which did not differ between strains (Figure S2). Data are shown as mean values ± S.E.M (n = 5 for each). * indicates significance (p < 0.05) using extra sum-of-squares F test.

**
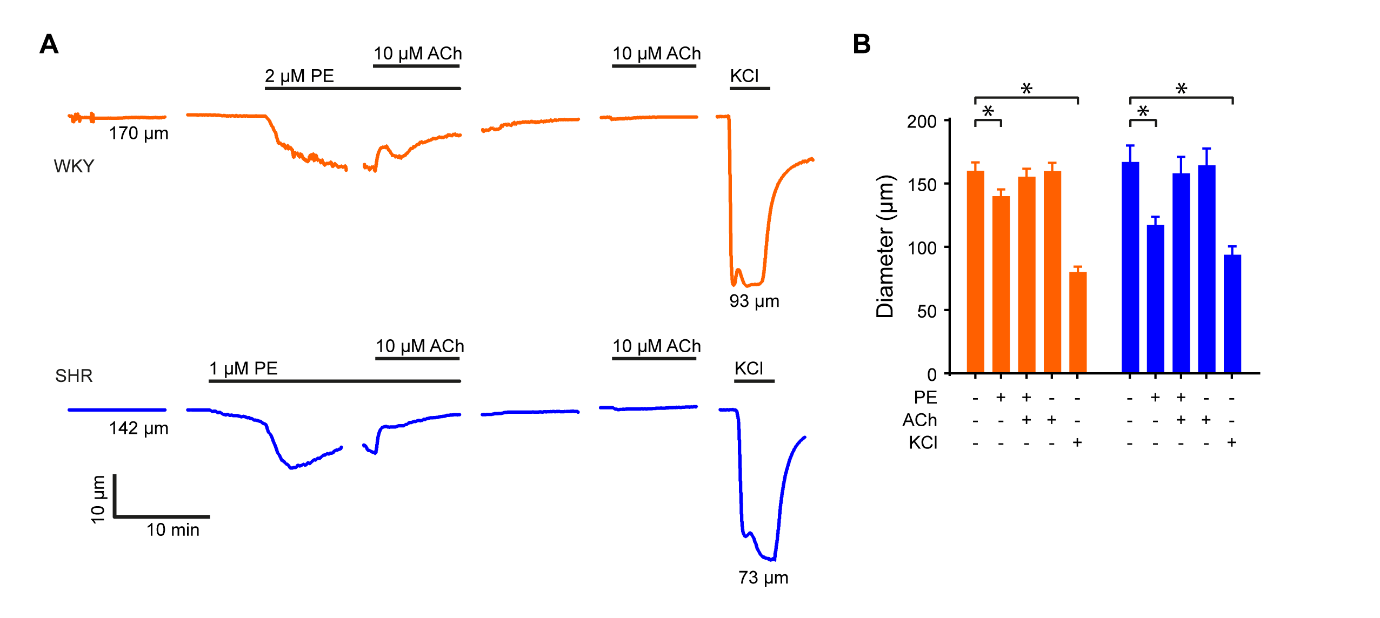
**

**Figure S5 – Acetylcholine evokes relaxation, not contraction, of small mesenteric arteries.** A) Representative artery diameter traces from normotensive (WKY, top) and hypertensive (SHR, bottom) animals showing the effect of ACh (10 µM) in the presence of PE (contracted artery) and in its absence (quiescent artery). Arteries of SHR were activated with 1 µM PE. However, some WKY arteries failed to respond to 1 µM PE. In these experiments, the PE concentration was increased to 2 µM to evoke contraction. B) Summary of diameter data comparing the responses of WKY and SHR rat arteries to PE, ACh and KCl, as indicated. Data are shown as mean values ± S.E.M (n = 5 for each). * indicates p < 0.05, repeated measures ANOVA with Sidak’s multiple comparisons test.


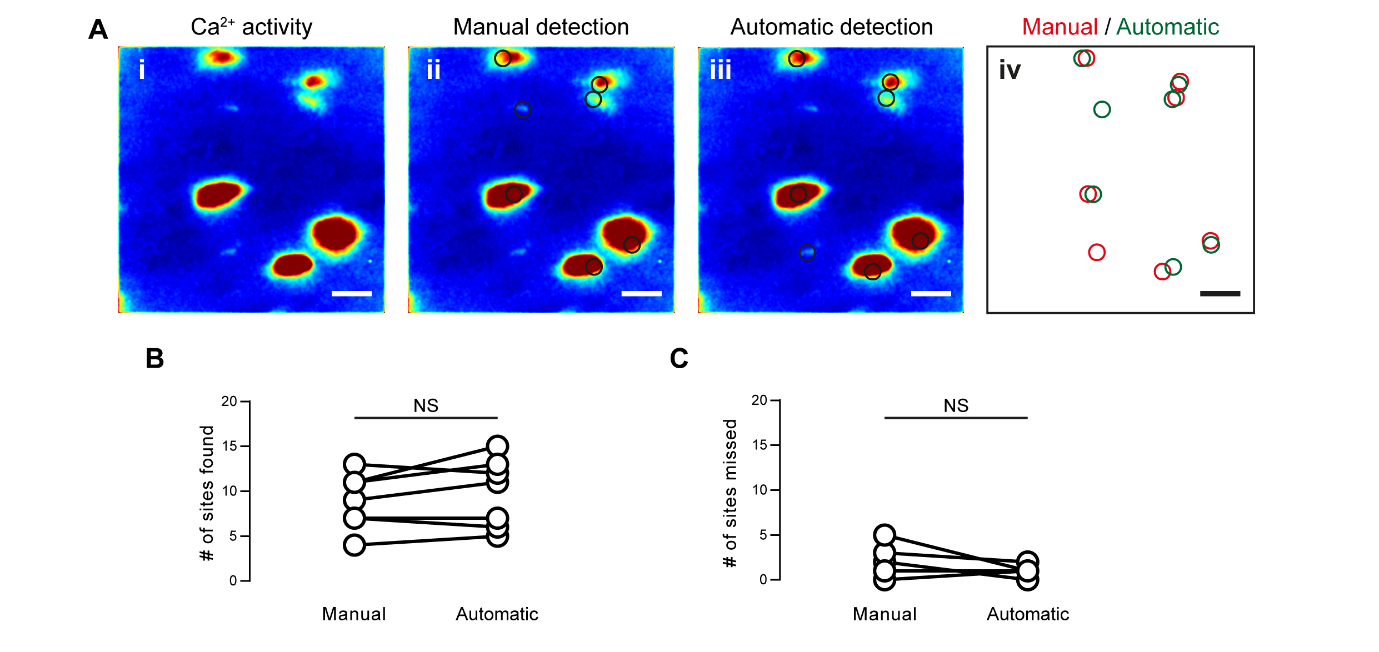


**Figure S6 – Comparison of manual and automated identification of Ca^2+^ event initiation sites.** A) Composite images showing (i) the standard deviation of intensity from one-minute recordings of endothelial Ca^2+^ activity with Ca^2+^ event initiation sites marked by ROIs obtained by manual (ii) and automated (iii) methods, and an image showing the ROIs overlaid (iv). Manual identification of Ca^2+^ event initiation sites, by an experienced researcher, was performed by scrolling through Δ*F* image stacks and placing ROIs at the location where a Ca^2+^ event was judged to have arisen from. Automated analysis was performed as described in the methods section. In this example, each method failed to detect one initiation site (that was detected by the other method) from which a small Ca^2+^ event arose. Scale bars = 20 µm. B-C) Summary data illustrating the number of Ca^2+^ event initiation sites found (B) and missed (C) by manual and automated identification methods.


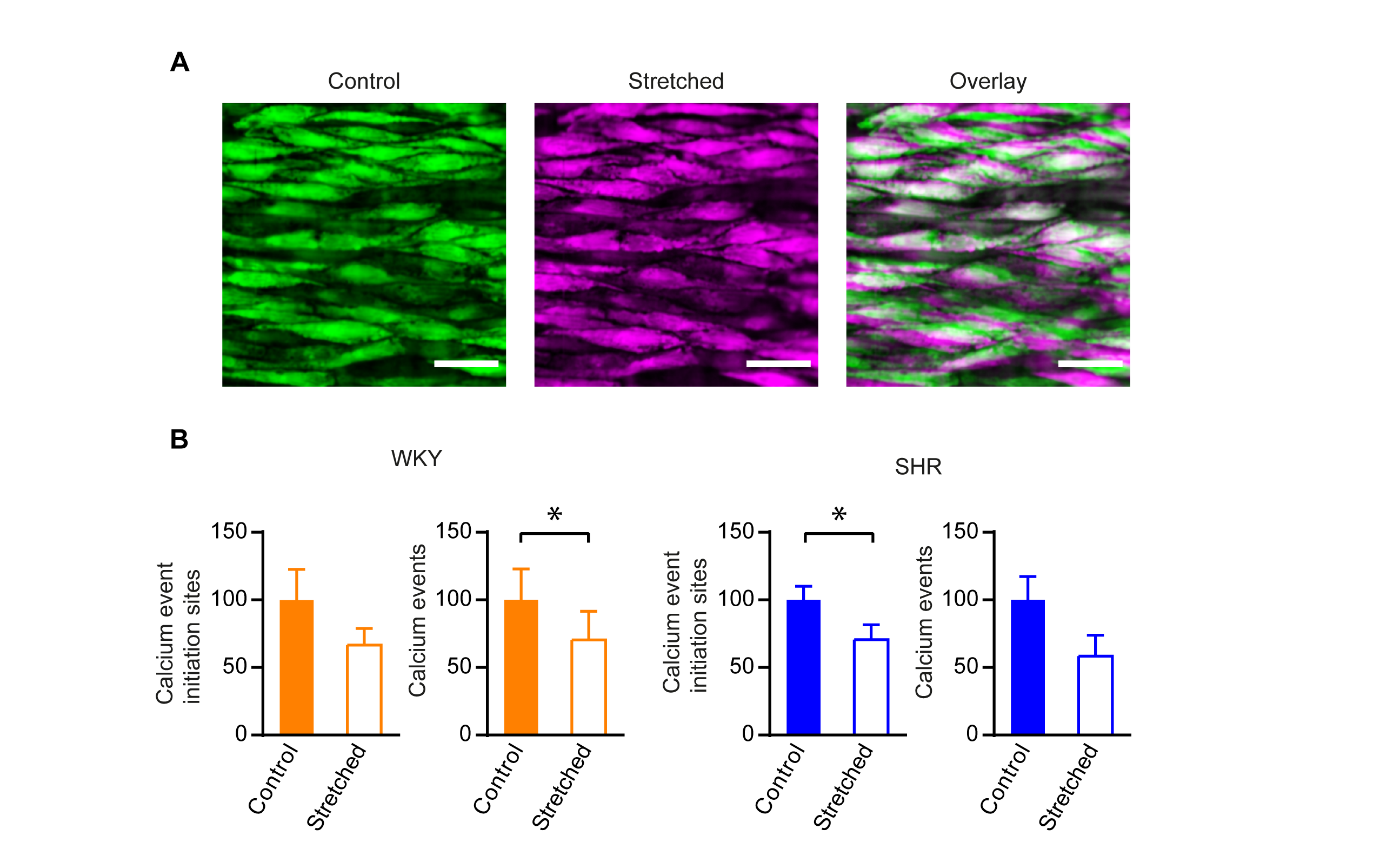


**Figure S7 – Endothelium-dependent reduction of vascular tone is impaired by mechanical stretch.** A) Representative Ca^2+^ images showing the same field of endothelial cells (left), before and after an increase in circumferential stretch of ~ 10% (middle), and an composite overlay of the two images (right). Scale bars = 20 µm. B) Summary of basal Ca^2+^ signaling data illustrating the effects of stretch on the number of event initiation sites and the total number of events. Data are shown as mean values ± S.E.M (n = 4 for each). * indicates significance (p < 0.05) using paired t-test.


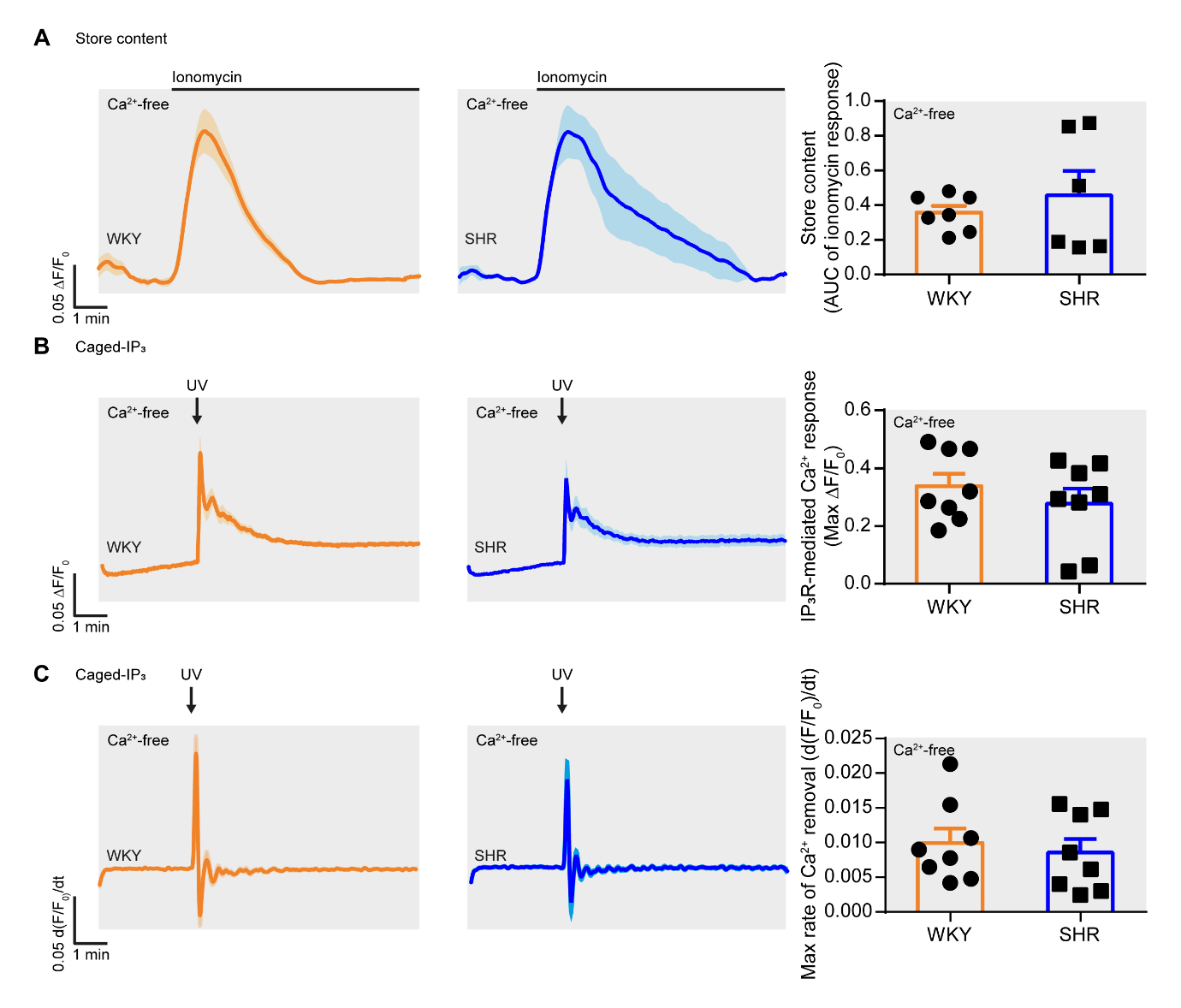


**Figure S8 – Ca^2+^ store content, IP_3_-mediated Ca^2+^ release, and Ca^2+^ removal is unaltered in hypertension.** A) Ca^2+^ responses to ionomycin in WKY (left) and SHR (middle) endothelial cell. Experiments were performed in the absence of external Ca^2+^. Each trace is the mean ± S.E.M of the global endothelial Ca^2+^ response from 8 biological replicates. The right panel is summary data indicating the total releasable pool of Ca^2+^ (area under the curve of ionomycin trace) in each strain. B) Ca^2+^ responses to photolysis of caged IP_3_ in WKY (left) and SHR (middle) endothelial cell. Experiments were performed in the absence of external Ca^2+^. Each trace is the mean ± S.E.M of the global endothelial Ca^2+^ response from 8 biological replicates. The right panel is summary data indicating the ability of IP_3_ to evoke Ca^2+^ release (max ΔF/F_0_ of response to photolysis of caged IP_3_). of ionomycin data indicating the total releasable pool of Ca^2+^ (area under the curve of ionomycin trace) in each strain. C) Differentiated Ca^2+^ responses to photolysis of caged IP_3_ (original F/F_0_ data shown in B), showing the rate of change of intracellular Ca^2+^ concentration in WKY (left) and SHR (right) endothelium. Each trace is the mean ± S.E.M of the global endothelial Ca^2+^ response from 8 biological replicates. The right panel is summary data indicating the maximum rate of Ca^2+^ removal (minima of derivate Ca^2+^ trace) in each strain.

**Supplementary Movie Legends**

**Movie S1: Assessing vascular reactivity in en face artery preparations.** The left panel shows a typical contraction of an en face artery to perfusion of phenylephrine (1 µM), and a subsequent dilation upon the addition of acetylcholine (1 µM). The superimposed blue lines indicate the width (unfolded circumference) of the flattened artery preparation. The right panel shows the time course of the mechanical response (average width of the artery, converted to equivalent diameter).

**Movie S2: Spontaneous calcium signaling in normotensive endothelium.** Panels in the top row show: left, a raw recording of endothelial Ca^2+^ activity; middle, the same data represented as a pseudocoloured fractional increases in Ca^2+^ (ΔF/F_0_); and right, automatically detected Ca^2+^ events (white) with circular ROIs placed at the site of initiation. The bottom panel show traces from each of the circular ROIs. Data also shown in Fig 3A.

**Movie S3: Spontaneous calcium signaling in hypertensive endothelium.** Panels in the top row show: left, a raw recording of endothelial Ca^2+^ activity; middle, the same data represented as a pseudocoloured fractional increases in Ca^2+^ (ΔF/F_0_); and right, automatically detected Ca^2+^ events (white) with circular ROIs placed at the site of initiation. The bottom panel show traces from each of the circular ROIs. Data also shown in Fig 3B.
