# Supplementary figures and images for: Increased Vascular Contractility in Hypertension Results From Impaired Endothelial Calcium Signaling
Source: Hypertension. 2019 Sep 23;74(5):1200–14. doi: 10.1161/HYPERTENSIONAHA.119.13791 (PMC6791503; doi:10.1161/HYPERTENSIONAHA.119.13791)

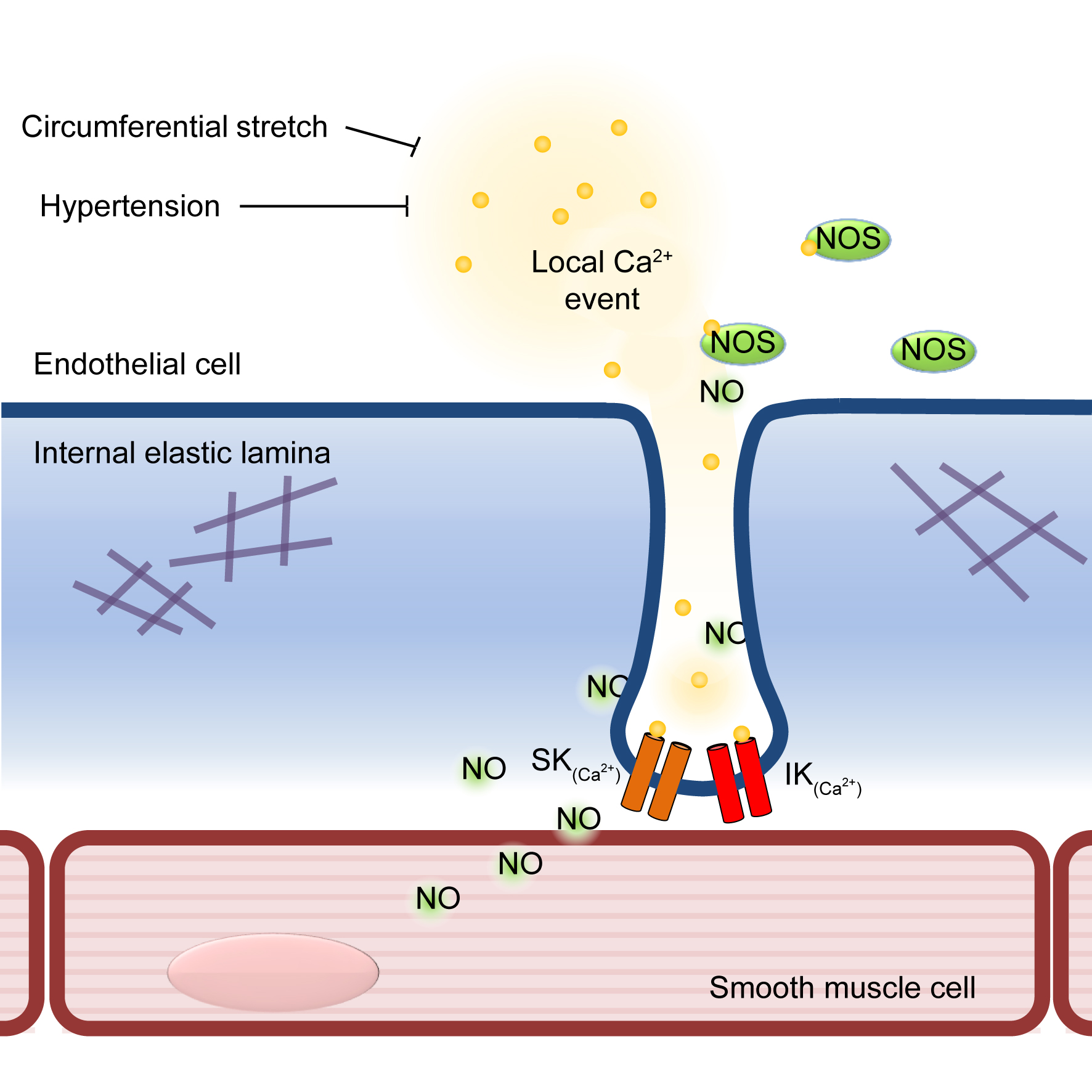

Supplement: Supplementary file 2 [file hyp-74-1200-s002.jpeg]
